# On hybrid nanofluid Yamada-Ota and Xue flow models in a rotating channel with modified Fourier law

**DOI:** 10.1038/s41598-021-98306-z

**Published:** 2021-10-01

**Authors:** Muhammad Ramzan, Hina Gul, M. Y. Malik, Dumitru Baleanu, Kottakkaran Sooppy Nisar

**Affiliations:** 1 Department of Computer Science, Bahria University, Islamabad, 44000 Pakistan; 2 Department of Mathematics, College of Sciences, King Khalid University, Abha, 61413 Saudi Arabia; 3 Department of Mathematics, Cankaya University, Ankara, 06790 Turkey; 4 Institute of Space Sciences, 077125 Magurele, Bucharest, Romania; 5 Department of Medical Research, China Medical University Hospital, China Medical University, Taichung, 40447 Taiwan; 6 Department of Mathematics, College of Arts and Sciences, Prince Sattam Bin Abdulaziz University, Wadi Aldawaser, 11991 Saudi Arabia

**Keywords:** Software, Mechanical engineering

## Abstract

The present study analyzes the comparison of the Xue and Yamada-Ota models for a hybrid nanoliquid flow in porous media occurring amidst a rotating channel with surface catalyzed reaction. Here, the hybrid nanofluid flow is studied under the effect of Cattaneo Christov (C–C) heat flux and homogenous heterogeneous (Homo-Hetero) chemical reaction with entropy generation minimization analysis. The assumptions of the viscosity of hybrid nanomaterial fluid and variable thermal conductivity are added characteristics to the inimitability of the flow model. Two kinds of nanoparticles, namely single-wall carbon nanotubes and multi-wall carbon nanotubes with ethylene glycol (EG) as the base fluid are considered. Carbon nanotubes possess diverse applications in daily life including energy storage, drug delivery, cancer treatment, tissue generation, platelet activation, magnetic force microscopy, and microwave absorption, etc. Similarity transformations are utilized to translate the modeled problem into the coupled ordinary differential equations. This system of ordinary differential equations is addressed numerically. The graphical outcomes are scrutinized by utilizing the MATLAB software bvp4c function. The results revealed that the velocity profile decreases for the higher rotation parameter while increases for the escalated slip parameter. Furthermore, the fluid concentration and temperature are on the decline for higher surface catalyzed reaction and thermal relaxation parameters respectively.

## Introduction

Carbon nanotubes (CNTs) were first time devised in 1991. CNTs possess a diameter of 0.7–50 nm, are thin cylinders formed of pure carbon. The role of CNTs is essential in numerous modern applications consist of composite materials, nanotechnology, conductive plastics, and atomic force microscope, etc. Two famous types of CNTs, i.e., single walls (SWCNTs) and multi-wall (MWCNTs) are identified in the literature. Significant importance is given by the researchers centering CNTs. Acharya et al.^1^ introduced the rotating MHD flow amalgamated with CNTs past a stretching surface. To construct the numerical solution of the non-linear flow problem, the RK-4 procedure is adopted. It is comprehended that for large radiation parameter estimates, the thermal profile increases. Unsteady squeezing CNTs immersed MHD nanofluid flow with viscous dissipation, and entropy generation in a rotating channel is inspected by Dawar et al.^2^. Kumar et al.^3^ explored the combined impacts of heat radiation and Hall current on a 3D Micropolar nanoliquid flow with submerged CNTs between two rotating sheets. The physical model characteristics of heat transfer and CNTs immersed nanoliquid flow in an asymmetric permeable channel are studied by Pandit et al.^4^. The wavelet collocation process is used for numerical results. The characteristics of CNTs nanoliquid squeezing flow in three dimensional with the lower stretching wall in a rotating channel is investigated numerically by Khan et al.^5^. The numerical technique known as the Runge–Kutta-Fehlberg (RKF) method to address the resulting equations is engaged here. Lately, the flow of the nanofluid with submersed CNTs in varied geometries may be found in^6–10^.

It is a very much understood reality that the transfer of heat phenomenon occurs owing to temperature inconsistency amidst two distinct objects or within an entity. For almost one century, the Fourier law (conduction of heat)^11^ was taken as a general principle for heat transfer processes. But later it is realized that in Fourier law if an issue occurs at the beginning that will carry out throughout the entire process and it opposes the causality principle. To answer this matter Cattaneo^12^ implanted the thermal relaxation time term in the typical Fourier's law which empowers the transfer of heat by the method of propagation of thermal waves with controlled speed and it was difficult to obtain a single heat equation. Later on, Christov updated the Cattaneo model and got a single equation for the temperature field. This improved model^13^ is categorized as the Cattaneo-Christov (C–C) heat flux model. Thermal stratification and C–C heat flux with base fluid EG and immersed CNTs nanofluid flow in rotating frame discussed by Ramzan et al.^14^. The bvp4c MATLAB technique for a numerical solution is used here. It is noticed that the thermal profile reduces for greater stratification parameter. Hayat et al.^15^ introduced C–C heat flux squeezed flow in a rotating frame. The series solutions for temperature and velocity distributions are formed by using the Homotopy Analysis Method. Karim and Samad^16^ discussed the influence of Brownian diffusion with C–C heat flux on elastic viscous squeezing nanoliquid flow and double slip impact in a channel. Some latest attempts about nanoliquid can be witnessed through^17–21^.

Hybrid nanoliquid, is the developing field of engineering that has trapped the eye of copious researchers who were looking at ways to enhance the productivity of cooling processes in the industry. Nanoliquid is a fluid that is made by the propagation of strong particles with sizes less than 100 nm in fluids. A nanoliquid with low thermal conductivity is one of its striking parameters that can restrict the performance of the heat transfer. Due to this shortcoming, normal heat transport fluids such as H_2_O and (CH_2_OH)_2_, and motor oil have limited heat transfer capabilities and are therefore unable to meet today’s cooling requirements. Suspended nanoparticles can improve the fluid flow and heat transfer features of the base liquid. Extensive studies are conducted by the researchers to highlight numerous aspects of nanofluid flows. Gul et al.^22^ examined nanoliquid flow in a rotating channel with C–C heat flux containing CNTs with (CH_2_OH)_2_ as a base fluid. Ramzan et al.^23^ investigated the nanoliquid flow with the effect of C–C heat flux under the influence of the H–H reaction. Hayat et al.^24^ introduced nanoliquid with CNTs and Darcy-Forchheimer. They utilized water as a base fluid. Ramzan et al.^25^ initiated the C–C impact on Tangent hyperbolic liquid flow amalgamated with second-order slip. They utilized the Runge–Kutta Fehlberg technique to tackle the problem. Some more explorations about nanoliquid may be found in^26–32^.

The aforementioned literature review discloses that copious studies are available that focus on the flow of nanofluids with immersed CNTs in numerous geometries. Nevertheless, limited explorations may be quoted with hybrid nanoliquid flows with engrossed CNTs and EG as a customary liquid. But no study until now is conducted that deliberated the comparative analysis of hybrid nanofluid models *i.e.,* Xue and Yamada and Ota amidst a permeable rotating channel with C–C heat flux amalgamated with the Homo-Hetero reactions and surface catalyzed reaction. The surface catalyzed reaction triggers the chemical reaction in a comparatively lesser time. Furthermore, the fluid models discussed in the literature assumed constant viscosity and thermal conductivity but in real engineering applications, both are considered as a variable. Here, the uniqueness of the fluid model is also augmented by considering both impacts as a variable which makes this study more realistic. The numerical results of the problem are obtained by using the bvp4c MATLAB technique. Through this investigation, we intend to answer the following questions:

i. How fluid velocity is influenced by the rotation and the slip impacts?
ii. What are the impacts of the surface-catalyzed parameter and the thermal relaxation parameter on the fluid concentration and the temperature respectively?
iii. Which hybrid nanofluid model is more dominating?
iv. How entropy generation rate is affected by the porosity parameter and the Reynolds number?
v. What are the effects of the Prandtl and Schmidt numbers of the heat transfer and mass transfer rates respectively?

## Mathematical formulation

**Figure 1:**
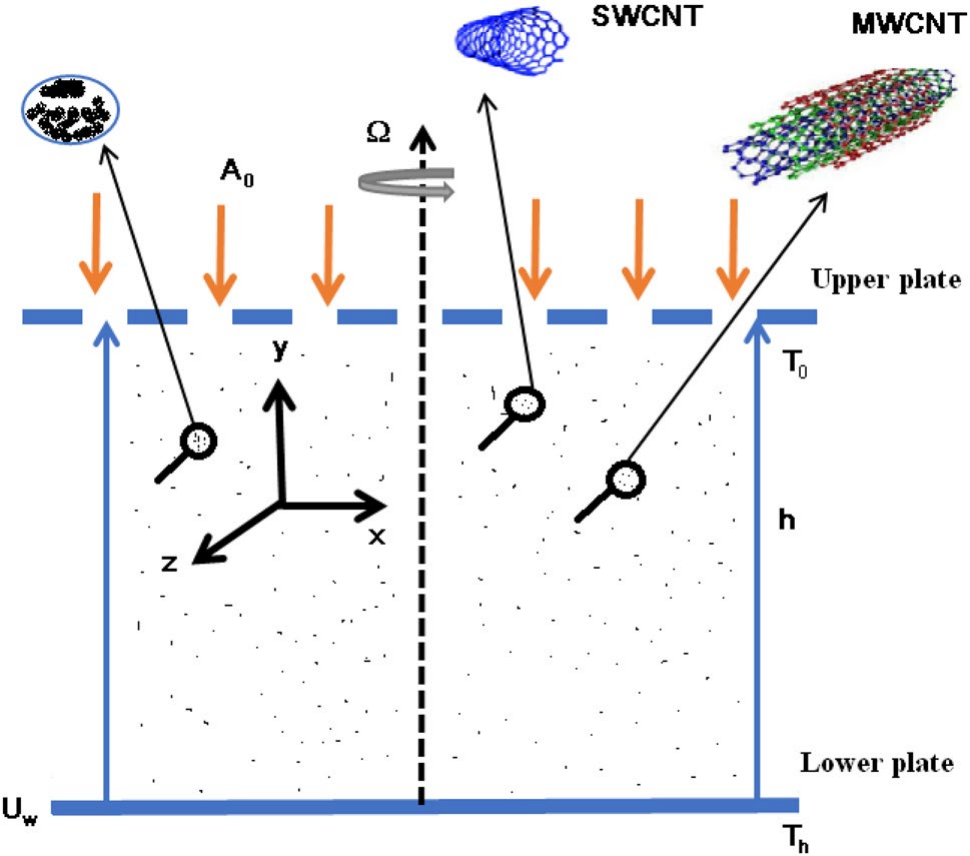
Flow problem^33^.

Assume the flow of hybrid nanofluid (SWCNTs-MWCNTs/EG) with Yamada-Ota and Xue models between two parallel plates. The whole system rotates about the *y-*axis with fixed angular velocity ($\Omega$_)_. The value of $\Omega$ specifies the direction of rotation of both lower and upper plates, that is for $\Omega$ > 0 both plates rotate in the same direction and for $\Omega$ < 0 both plates are rotated in the opposite direction while for static plates, we assign the value of $\Omega$ = 0. And both plates are distance “*h*” apart. The lower plate moves faster as compared to the upper plate with *U*_*w*_ = a*x* (a > 0). The positions of the upper and lower plates are at *y* = *h*, and *y* = *0*, respectively. The coordinate system is taken in such a way that plates are parallel to the *x-axis*, and the *y-axis* is perpendicular to the plates (Fig. 1).

For rotating flow, the momentum governing equation is^33–35^:

1 $$ \rho_{HNF} \left( {\frac{dV}{{dt}} + 2\Omega \times v + \Omega \times (\Omega \times r)} \right) = div\tau . $$

The governing boundary layer equations under the impact of C–C heat flux are represented as:

2 $$ u_{X} + v_{Y} = 0, $$

3 $$ \rho_{HNF} (uu_{X} + vu_{Y} + 2\Omega w) = - P_{X} + \mu_{HNF} (u_{XX} + u_{YY} ) - \frac{{\mu_{HNF} }}{{k^{*} }}u $$

4 $$ \rho_{HNF} (uv_{X} + vv_{Y} ) = - P_{Y} + \mu_{HNF} (v_{XX} + v_{YY} ), $$

5 $$ \rho_{HNF} (uw_{X} + vw_{Y} - 2\Omega u) = \mu_{HNF} (w_{XX} + w_{YY} ) - \frac{{\mu_{HNF} }}{{k^{*} }}w. $$

The modified pressure is defined as $P = p - \frac{{\Omega^{2} X^{2} }}{2}$. Mathematically the heat transfer phenomenon can be expressed as:

6 $$ (\rho c_{p} )_{HNF} (uT_{X} + vT_{Y} ) = - \nabla .{\mathbf{q}} + \frac{{Q_{0} (T - T_{0} )}}{{(\rho c_{p} )_{HNF} }}, $$

where ${\mathbf{q}}$ is the heat flux which satisfies the following relationship:

7 $$ {\mathbf{q}} + \lambda_{1} ({\mathbf{q}}_{t} - {\mathbf{q}}.\nabla V + V.\nabla {\mathbf{q}} + (\nabla .V){\mathbf{q}}) = - k_{HNF} (T)\nabla T. $$

Removing **q** from (6) and (7) following Hayat^15^, we get:

8 $$ \begin{aligned} uT_{X} + vT_{Y} & = \frac{\partial }{\partial y}\left( {\frac{{k_{HNF} (T)}}{{(\rho c)_{HNF} }}T_{Y} } \right) - \lambda_{2} (u^{2} T_{XX} + v^{2} T_{YY} + 2uvT_{XY} + (uu_{X} + vu_{Y} )T_{X} \\ & \quad + ((uv_{X} + vv_{Y} )T_{Y} ) + \frac{{Q_{0} (T - T_{0} )}}{{(\rho c_{p} )_{HNF} }}, \\ \end{aligned} $$

9 $$ ua_{X} + va_{Y} = \frac{\partial }{\partial y}\left( {D_{A} (a)a_{Y} } \right) - k_{1} ab^{2} - Sk_{s} a, $$

10 $$ ub_{X} + vb_{Y} = \frac{\partial }{\partial y}\left( {D_{B} (b)b_{Y} } \right) + k_{1} ab^{2} + Sk_{s} a. $$

The associated B. C’s are presented as:

11 $$ u = U_{w} + s_{1} \frac{{\mu_{HNF} }}{{\rho_{HNF} }}u_{Y} ,v = 0,w = 0, T = T_{h} + \chi_{1} \frac{{k_{HNF} (\widehat{T})}}{{k_{F} }}T_{Y} ,D_{A} a_{Y} = k_{s} a,\,D_{B} b_{Y} = - k_{s} a\,\,{\text{at}}\;y \, = \, 0, $$

12 $$ u = 0,v = - A_{0} ,w = 0,T = T_{\infty } ,\,\,\,a \to a_{0} ,b \to 0,\;{\text{as}}\;y \, \to \, h, $$

where $- A_{0}$ is represented the uniform suction/injection for injection $( - A_{0} < 0),$ and the suction $( - A_{0} > 0),$ velocity at the upper wall. The temperature-dependent thermal conductivity is specified as:

13 $$ K(T) = \kappa_{\infty } \left( {1 + \varepsilon_{1}^{{}} \frac{{T - T_{0} }}{{T_{h} - T_{0} }}} \right), $$

with $\kappa_{\infty }$ is the free stream thermal conductivity. Here, $\varepsilon_{1} > 0$ expressing for gas while liquid characteristics are represented by $\varepsilon_{1} < 0,$ with $\varepsilon_{1}$ is of unit dimension.

Also, $D_{A} \left( a \right)$ is the diffusion coefficient (concentration-dependent) of chemical species $A^{*}$, and $D_{B} \left( b \right)$ is the diffusion coefficient (concentration-dependent) of chemical specie $B^{*}$ and are defined as follows:

14 $$ D_{A} \left( a \right) = D_{A} \left[ {1 + \varepsilon_{2} \left( {\frac{a}{{a_{0} }}} \right)} \right], $$

15 $$ D_{B} \left( b \right) = D_{B} \left[ {1 + \varepsilon_{2} \left( {\frac{b}{{a_{0} }}} \right)} \right], $$

where $\varepsilon_{2}$ is of unit dimension.

Tables 1, 2, and 3 describe the thermophysical features of the hybrid nanofluid flow models, and CNTs/base fluid respectively.

**Table 1:** Thermophysical attributes of hybrid nanoliquid^34^.

Density	$$\begin{gathered} \rho_{HNF} = \rho_{F} \left( {1 - \phi_{2} } \right)\left( {(1 - \phi_{1} ) + \phi_{1} \left( {\frac{{\rho_{s1} }}{{\rho_{F} }}} \right)} \right) + \phi_{2} \rho_{{s_{2} }} ,\frac{{\rho_{HNF} }}{{\rho_{F} }} = A. \hfill \\ \hfill \\ \end{gathered}$$
Heat Capacity	$$\begin{aligned} & (\rho c_{p} )_{HNF} = \phi_{2} (\rho c_{p} )_{{s_{2} }} + (1 - \phi_{2} )(\rho c_{p} )_{F} \left\{ {\phi_{1} \frac{{(\rho c_{p} )_{{s_{1} }} }}{{(\rho c_{p} )_{F} }} + [(1 - \phi_{1} )]} \right\} \\ \,\,\,\,\,\,\,\,\,\,\,\,\,\,\,\,\,\,\,\,\,\,\,\,\,\,\,\,\,\,\,\,\,\,\,\,\,\, & \frac{{(\rho c_{p} )_{HNF} }}{{(\rho c_{p} )_{F} }} = C \\ \end{aligned}$$
Variable viscosity	$\mu_{HNF} = \frac{{\mu_{F} }}{{(1 - \phi_{1} )^{2.5} (1 - \phi_{2} )^{2.5} }},\,\,\,\,\,\frac{{\mu_{HNF} }}{{\mu_{F} }} = B.$
Thermal conductivity	$$\begin{aligned} \frac{{k_{HNF} (T)}}{{k_{bF} }} & = \frac{{(n - 1)k_{bF} + k_{{s_{2} }} - (k_{bF} - k_{{s_{2} }} )(n - 1)\phi_{2} }}{{(n - 1)k_{bF} + k_{{s_{2} }} + (k_{bF} - k_{{s_{2} }} )\phi_{2} }}(1 + \varepsilon_{1} \theta ) \\ \frac{{k_{bF} }}{{k_{F} }} & = \frac{{(n - 1)k_{F} + k_{{s_{1} }} - (n - 1)\phi_{1} (k_{F} - k_{{s_{1} }} )}}{{k_{F} (n - 1) + k_{{s_{1} }} + \phi_{1} (k_{F} - k_{{s_{1} }} )}},\,\,\,\frac{{k_{HNF} }}{{k_{bF} }} = E,\frac{{k_{bF} }}{{k_{F} }} = D \\ \end{aligned}$$
Xue-model	$$\begin{aligned} \frac{{k_{HNF} }}{{k_{bF} }} & = \frac{{1 - \phi_{2} + 2\phi_{2} \left( {\frac{{k_{{s_{2} }} }}{{k_{{s_{2} }} - k_{bF} }}} \right)\ln \frac{{k_{{s_{2} }} + k_{bF} }}{{2k_{bF} }}}}{{1 - \phi_{2} + 2\phi_{2} \left( {\frac{{k_{{s_{2} }} }}{{k_{{s_{2} }} - k_{bF} }}} \right)\ln \frac{{k_{{s_{2} }} + k_{bF} }}{{2k_{bF} }}}}, \\ \frac{{k_{bF} }}{{k_{F} }} & = \frac{{1 - \phi_{1} + 2\phi_{1} \left( {\frac{{k_{{s_{1} }} }}{{k_{{s_{1} }} - k_{F} }}} \right)\ln \frac{{k_{s1} + k_{F} }}{{2k_{F} }}}}{{1 - \phi_{1} + 2\phi_{1} \left( {\frac{{k_{F} }}{{k_{s1} -_{F} }}} \right)\ln \frac{{k_{s1} + k_{F} }}{{2k_{F} }}}}. \\ \\ \end{aligned}$$
Yamada-Ota model	$$\begin{aligned} \frac{{k_{HNF} }}{{k_{bF} }} & = \frac{{1 + \frac{{k_{bF} }}{{k_{{s_{2} }} }}\frac{L}{R}\phi_{2}^{0.2} + \left( {1 - \frac{{k_{bF} }}{{k_{{s_{2} }} }}} \right)\phi_{2} \frac{L}{R}\phi_{2}^{0.2} + 2\phi_{2} \left( {\frac{{k_{{s_{2} }} }}{{k_{{s_{2} }} - k_{bF} }}} \right)\ln \frac{{k_{{s_{2} }} + k_{bF} }}{{2k_{{s_{2} }} }}}}{{1 - \phi_{2} + 2\phi_{2} \left( {\frac{{k_{bF} }}{{k_{{s_{2} }} - k_{bF} }}} \right)\ln \frac{{k_{{s_{2} }} + k_{bF} }}{{2k_{bF} }}}}, \\ \frac{{k_{bF} }}{{k_{F} }} & = \frac{{1 + \frac{{k_{F} }}{{k_{{s_{1} }} }}\frac{L}{R}\phi_{1}^{0.2} + \left( {1 - \frac{{k_{F} }}{{k_{{s_{1} }} }}} \right)\phi_{1} \frac{L}{R}\phi_{1}^{0.2} + 2\phi_{1} \left( {\frac{{k_{{s_{1} }} }}{{k_{{s_{1} }} - k_{F} }}} \right)\ln \frac{{k_{{s_{1} }} + k_{F} }}{{2k_{{s_{1} }} }}}}{{1 - \phi_{1} + 2\phi_{1} \left( {\frac{{k_{F} }}{{k_{{s_{1} }} - k_{F} }}} \right)\ln \frac{{k_{{s_{1} }} + k_{F} }}{{2k_{F} }}}}. \\ \end{aligned}$$

**Table 2:** Thermophysical traits of Ethylene glycol and CNTs^14^.

Physical properties	Ethylene glycol	SWCNT	MWCNT
$\rho \left( {\frac{kg}{{m^{3} }}} \right)$	1115	2600	1600
$c_{p} \left( {\frac{J}{kg.K}} \right)$	2430	425	796
$K\left( {\frac{W}{m.K}} \right)$	0.253	6600	3000

**Table 3:** Numerical results of $C_{f} \sqrt {{\text{Re}}_{x} }$ for ${\text{Re}}$ and $\lambda$.

***Re***	${\varvec{\lambda}}$	$C_{f} \sqrt {{\text{Re}}_{x} }$	
		_Yamada-Ota model_	_Xue model_
1.5	0.5	3.2545	3.2526
2.5		3.2981	3.2965
3.5		3.3421	3.3405
	0.1	3.2036	3.2019
	0.4	3.2416	3.2401
	0.7	3.2803	3.2789

The Similarity transformations are characterized as:

16 $$ u = exf^{^{\prime}} (\eta ),\;v = - ehf(\eta ),\;v = - ehf(\eta ),\;\theta (\eta ) = \frac{{T - T_{0} }}{{T_{h} - T_{0} }},\;\phi = \frac{a}{{a_{0} }},H = \frac{b}{{a_{0} }},\;\eta = \frac{y}{h}. $$

By invoking the above transformation into the Eqs. (3) - (12), we obtain the following dimensionless equations to obtain the flow profiles.

17 $$ f^{iv} + BA{\text{Re}} (f^{\prime } f^{\prime \prime } - ff^{\prime \prime \prime } ) - A\Omega g^{\prime } - \lambda f^{\prime } = 0, $$

18 $$ g^{\prime\prime} - AB{\text{Re}} (gf^{\prime} - fg^{\prime}) + 2BA\Omega f^{\prime} + \lambda g = 0, $$

19 $$ \begin{gathered} \frac{ED}{C}\left( {\left( {1 + \varepsilon_{1} \theta } \right)\theta^{\prime\prime} + \varepsilon_{1} \theta^{{\prime}{2}} } \right) + \Pr {\text{Re}} (\frac{Q}{C}\theta + f\theta^{\prime} - \gamma (f^{2} \theta^{\prime\prime} + ff^{\prime}\theta^{\prime}) = 0, \hfill \\ \hfill \\ \end{gathered} $$

20 $$ \begin{gathered} (1 + \varepsilon_{2} \phi )\phi ^{\prime\prime} + \phi ^{{\prime}{2}} \varepsilon_{2} + {\text{Re}} Sc(f\phi ^{\prime} - k\phi H^{2} - K_{vs} \phi ) = 0, \hfill \\ \hfill \\ \end{gathered} $$

21 $$ (1 + \varepsilon_{2} \phi )H^{\prime\prime} + H^{{\prime}{2}} \varepsilon_{2} + {\text{Re}} \frac{Sc}{\delta }(f\,H^{\prime} + k\phi H^{2} + K_{vs} \phi ) = 0, $$

22 $$ \begin{aligned} f^{\prime}\left( 0 \right) & = 1 + S\frac{B}{A}f^{\prime\prime}(0),\,f\left( 0 \right){ = }0, g(0) = 0,\,\theta (0) = 1 + M\theta^{\prime}(0)\frac{{k_{HNF} }}{{k_{F} }}\left( {1 + \varepsilon_{1} \theta } \right), \\ (1 + \varepsilon_{2} \phi )\phi ^{\prime}(0) & = K_{s} \phi (0),\,\,\,\left[ {1 + \varepsilon_{2} H(0)} \right]H^{\prime}(0) = - K_{s} \phi (0), \\ f^{\prime}(h) & = 0,\,f(h) = \varepsilon ,\,g\,\,(h) = 0,\,\theta \,(h) = 0,\phi (h) = 1,H(h) = 0. \\ \end{aligned} $$

The quantities in the above equations are defined as:

23 $$ \begin{aligned} \gamma & = \lambda_{2} e,k = \frac{{k_{1} a_{0}^{2} }}{e},\;{\text{Re}} = \frac{{eh^{2} }}{{v_{F} }},Q = \frac{{Q_{0} }}{{c\left( {\rho c_{p} } \right)_{F} }},\varepsilon = \frac{{A_{0} }}{eh},\Pr = \frac{{\mu_{F} c_{p} }}{{k_{F} }},\;\Omega = \frac{{\gamma h^{2} }}{{\nu_{F} }},\;M = \frac{{\chi_{1} }}{h},\;S = \frac{{s_{1} \nu_{F} }}{h}, \\ K_{vs} & = S_{v} K_{s} ,K_{s} = \frac{{k_{s} \sqrt {\nu_{F} } }}{{D_{A} \sqrt e }}S_{v} = \frac{{SD_{A} }}{e},\,\,\,\delta = \frac{{D_{B} }}{{D_{A} }},\,\,Sc = \frac{{\nu_{F} }}{{D_{A} }},\,\,\,K_{s} = \frac{{k_{s} a_{0} }}{{D_{B} }},\,\,\,\,\,\, \\ \end{aligned} $$

We assume the diffusion coefficients of both chemical species are identical since their sizes are comparable. The following relationship will result from this assumption:

24 $$ H(\eta ) + \phi (\eta ) = 1, $$

25 $$ 2(1 + \varepsilon_{2} \phi )\phi ^{\prime\prime} + 2\varepsilon {}_{2}\phi ^{{\prime}{2}} + {\text{Re}} Sc(2f\phi ^{\prime} - K\phi (1 - \phi )^{2} - K_{vs} \phi = 0, $$

26 $$ \,\,\,\,\,\,\,\,\,\,\,\,\,\,\,\,\,\,\,\,\,\,\,\,\,\,\,\,\,\,(1 + \varepsilon_{2} \phi )\phi ^{\prime}(0) = K_{s} \phi (0),\,\,\,\phi (h) = 1. $$

Drag force coefficient, mass transfer rate, and Nusselt number are defined by:

27 $$ C_{f} = \frac{{2\tau_{w} }}{{\rho_{HNF} U_{w}^{2} }},\;Sh_{x} = \frac{{hj_{w} }}{{D_{B} (C_{w} - C_{\infty } )}},\;Nu_{r} = \frac{{hq_{w} }}{{k_{\infty } (T_{w} - T_{\infty } )}}, $$

with

28 $$ \tau_{w} = \mu_{HNF} \left( {\frac{\partial u}{{\partial y}}} \right)_{y = 0} ,q_{w} = - K(T)\left. {T_{y} } \right|_{y = 0} ,j_{w} = - D_{B} b_{y = 0} . $$

Dimensionless surface drag coefficient, mass transfer rate, and heat transfer rate are:

29 $$ C_{f} \sqrt {{\text{Re}}_{x} } = \left. {\left( {\frac{1}{{(1 - \varphi_{1} )^{2.5} (1 - \varphi_{2} )^{2.5} }}} \right)f^{\prime\prime}(\eta )} \right|_{\eta = 0} ,Sh_{x} = - \phi ^{\prime}(0),Nu_{x} = - (1 + \varepsilon \theta )\theta ^{\prime}(0). $$

## Entropy generation analysis

The volumetric entropy generation is represented as^36^:

30 $$ \begin{aligned} S_{GEN}^{\prime \prime \prime } & = \frac{{k_{HNF} }}{{T_{0}^{2} }}T_{Y}^{2} + \frac{{\mu_{HNF} }}{{T_{0} }}u_{Y}^{2} + \frac{{\mu_{HNF} }}{{T_{0} k*\Omega_{1} }}u^{2} + \frac{{RD_{A} (a)}}{{T_{0} }}a_{Y} T_{Y} \\ & \quad + \frac{{RD_{A} (a)}}{{a_{0} }}(a_{Y} )^{2} + \frac{{RD_{B} (b)}}{{T_{0} }}b_{Y} T_{Y} + \frac{{RD_{B} (b)}}{{a_{0} }}(b_{Y} )^{2} . \\ \end{aligned} $$

The entropy generation in the non-dimensional form is appended below:

31 $$ N_{S} = \frac{{S_{GEN}^{\prime \prime \prime } }}{{S_{0}^{\prime \prime \prime } }} = \left( \begin{gathered} \Omega_{2} ED\left( {1 + \varepsilon \theta } \right)\left( {\theta^{\prime } (\eta )} \right)^{2} + BBr(f^{\prime \prime 2} (\eta ) + \lambda *f^{\prime 2} (\eta ))  \hfill \\ \quad + L_{1} \left( {1 + \varepsilon_{2} \phi } \right)\phi^{\prime } \theta^{\prime } + \frac{{L_{1} }}{{\Omega_{2} }}\phi^{\prime 2} + L_{2} \left( {1 + \varepsilon_{2} (1 - \phi } \right)\phi^{\prime } \theta ^{\prime} + \frac{{L_{2} }}{{\Omega_{2} }}\phi^{\prime 2} \hfill \\ \end{gathered} \right), $$

where

32 $$ S_{0}^{\prime \prime \prime } = \frac{{k_{F} \nabla T}}{{h^{2} T_{0} }},\;Br = \frac{{\mu_{F} U_{w}^{2} h^{2} }}{{k_{F} (T_{h} - T_{0} )}},\,\,\Omega_{2} = \frac{{T_{h} - T_{0} }}{{T_{0} }},\,\lambda * = \frac{{\nu_{F} }}{{k^{*} \Omega_{1} }},\,\,L_{1} = \frac{{RD_{A} a_{0} }}{{k_{F} }},\,L_{2} = \frac{{RD_{B} a_{0} }}{{k_{F} }}. $$

The Bejan number (*Be*) is presented as:

33 $$ Be = \frac{{\left( {\frac{{k_{HNF} }}{{T_{0}^{2} }}T_{Y}^{2} + \frac{{RD_{A} (a)}}{{T_{0} }}a_{Y} T_{Y} + \frac{{RD_{A} (a)}}{{a_{0} }}(a_{Y} )^{2} + \frac{{RD_{B} (b)}}{{T_{0} }}b_{Y} T_{Y} + \frac{{RD_{B} (b)}}{{a_{0} }}(b_{Y} )^{2} } \right)}}{{\left( {\frac{{k_{HNF} }}{{T_{0}^{2} }}T_{Y}^{2} + \frac{{\mu_{HNF} }}{{T_{0} }}u_{Y}^{2} + \frac{{\mu_{HNF} }}{{T_{0} k*\Omega_{1} }}u^{2} + \frac{{RD_{A} (a)}}{{T_{0} }}a_{Y} T_{Y} + \frac{{RD_{A} (a)}}{{a_{0} }}(a_{Y} )^{2} + \frac{{RD_{B} (b)}}{{T_{0} }}b_{Y} T_{Y} + \frac{{RD_{B} (b)}}{{a_{0} }}(b_{Y} )^{2} } \right)}}. $$

In the dimensionless form, the *Be* is given as:

34 $$ Be = \frac{{\left( {\Omega_{2} ED\left( {\theta ^{\prime}(\eta )} \right)^{2} + L_{1} \left( {1 + \varepsilon_{2} \phi } \right)\phi^{\prime}\theta ^{\prime} + \frac{{L_{1} }}{{\Omega_{2} }}\phi ^{{\prime}{2}} + L_{2} \left( {1 + \varepsilon_{2} (1 - \phi )} \right)\phi ^{\prime}\theta ^{\prime} + \frac{{L_{2} }}{{\Omega_{2} }}\phi ^{{\prime}{2}} } \right)}}{{\left( \begin{gathered} \Omega_{2} ED\left( {\theta ^{\prime}(\eta )} \right)^{2} + CBr(f^{^{\prime\prime}2} (\eta ) + \lambda *f^{^{\prime}2} (\eta )) + L_{1} \left( {1 + \varepsilon_{2} \phi } \right)\phi^{\prime}\theta ^{\prime} + \frac{{L_{1} }}{{\Omega_{2} }}\phi ^{{\prime}{2}} \hfill \\ + L_{2} \left( {1 + \varepsilon_{2} (1 - \phi )} \right)\phi ^{\prime}\theta ^{\prime} + \frac{{L_{2} }}{{\Omega_{2} }}\phi ^{{\prime}{2}} \hfill \\ \end{gathered} \right)}}. $$

For a similar solution, the Brinkman number is given by:

35 $$ Br = \frac{{\mu_{F} U_{w}^{2} h^{2} }}{{k_{F} (T_{h} - T_{0} )}} = \frac{{\mu_{F} x^{2} e^{2} h^{2} }}{{k_{F} \left( {T_{h} - T_{0} } \right)}}. $$

To get rid of *x* in *Br,* the temperature $T_{h} (x)$ may be in the form $T_{h} = T_{0} + T_{1} x^{2} ,$ where $T_{1}$ is a constant, Else, the solution found is only locally similar. By utilizing the above-described transformation, Eq. (35) takes the form $Br = \frac{{\mu_{F} e^{2} h^{2} }}{{k_{F} T_{1} }}$.

## Results with discussion

**Figure 2:**
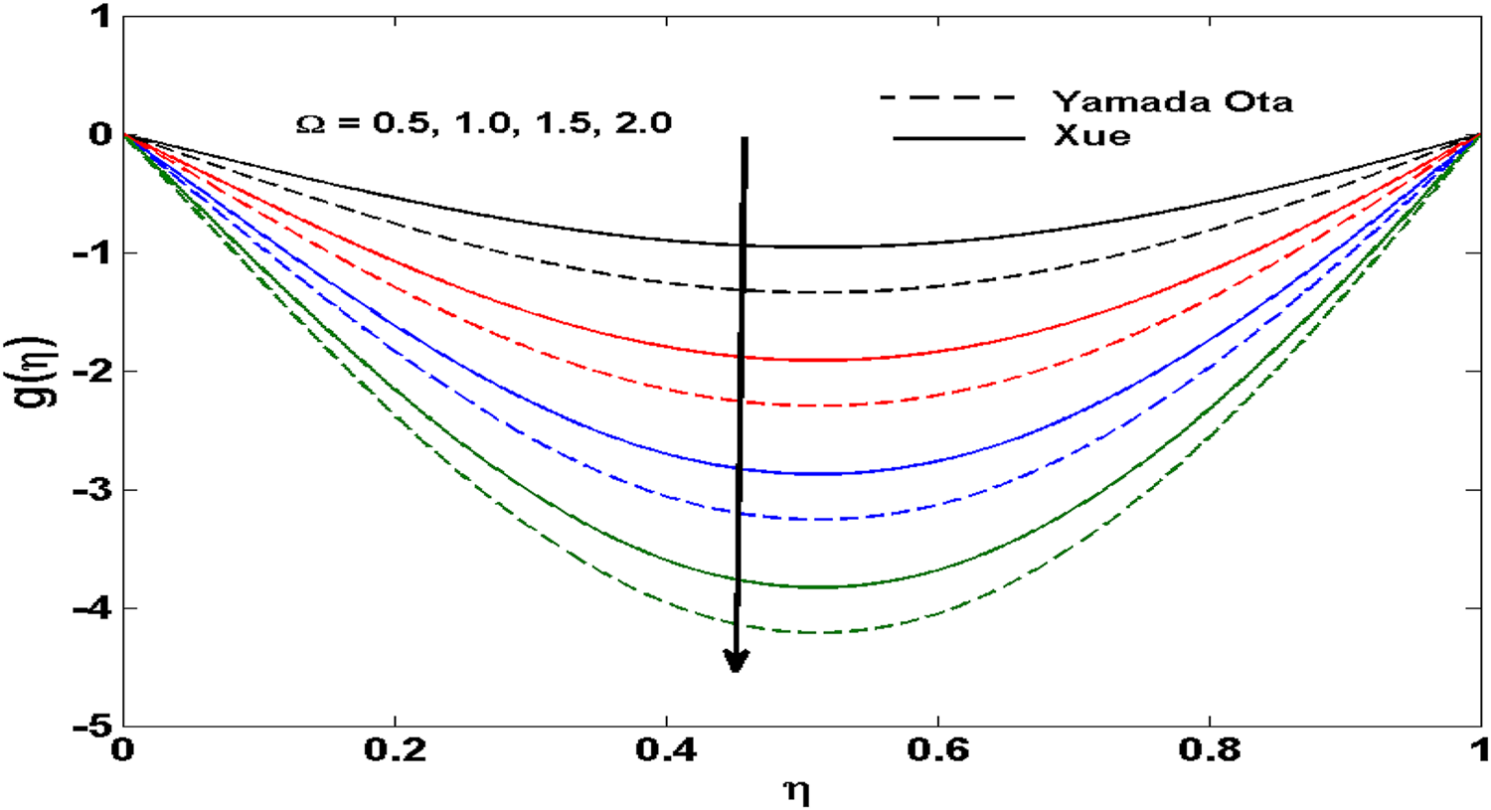
Behaviour of $g(\eta )$ vs $\Omega$. Image generated by using MATLAB 2015a https://www.mathworks.com/help/simulink/release-notes-R2015a.html.

**Figure 3:**
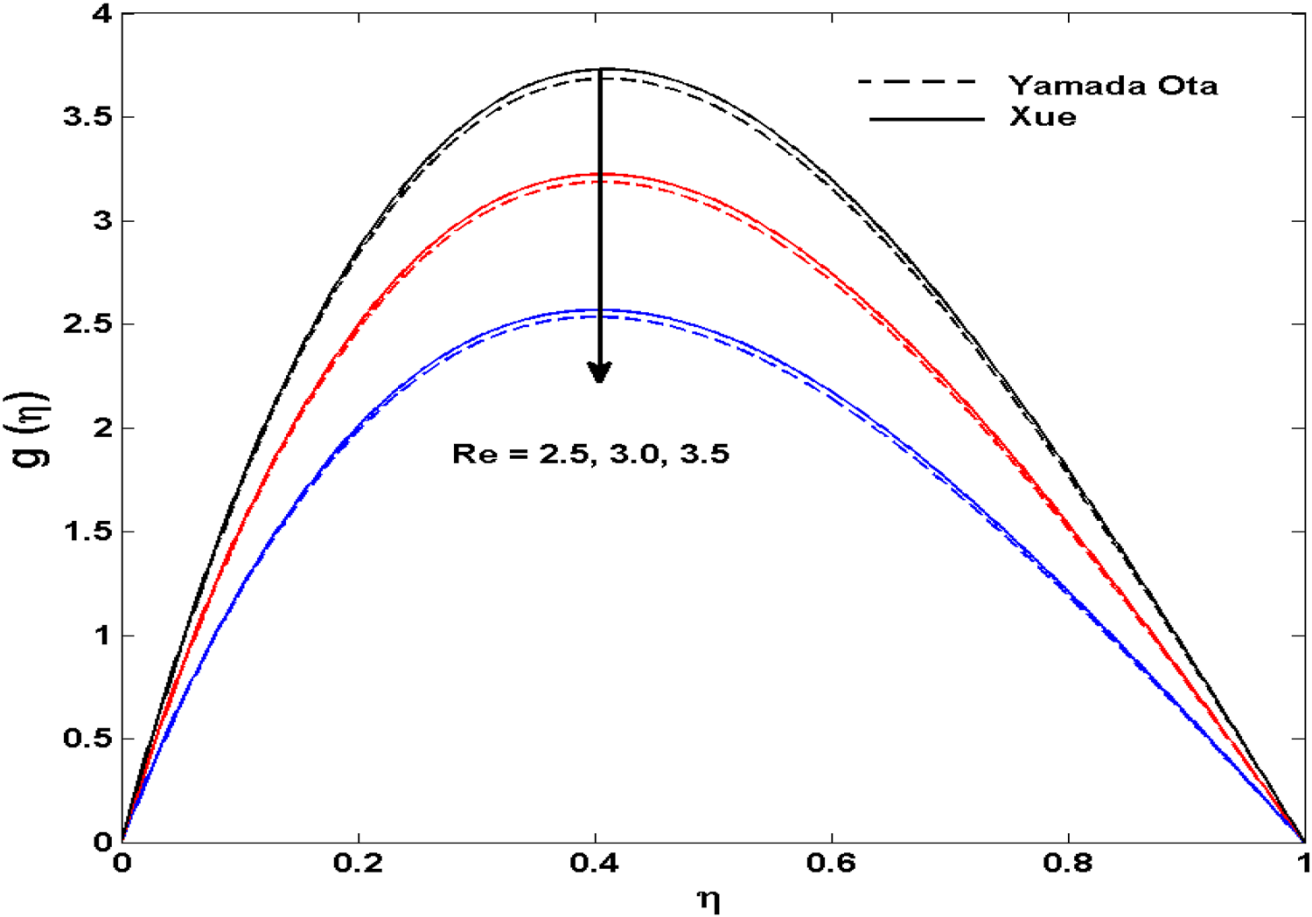
Behaviour of $g(\eta )$ vs ${\text{Re}}$. Image generated by using MATLAB 2015a https://www.mathworks.com/help/simulink/release-notes-R2015a.html.

**Figure 4:**
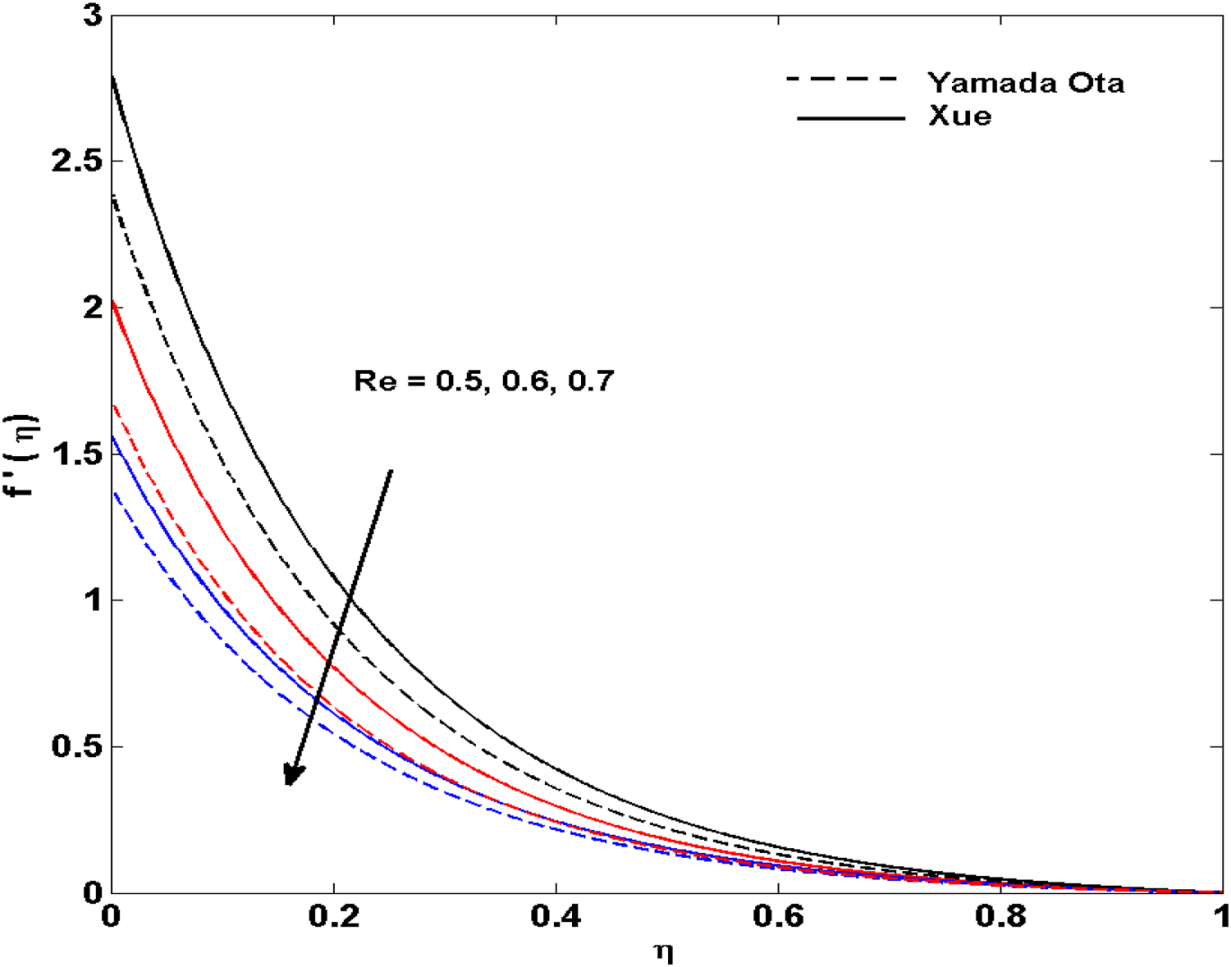
Behaviour of $f^{\prime}(\eta )$ vs ${\text{Re}}$. Image generated by using MATLAB 2015a https://www.mathworks.com/help/simulink/release-notes-R2015a.html.

**Figure 5:**
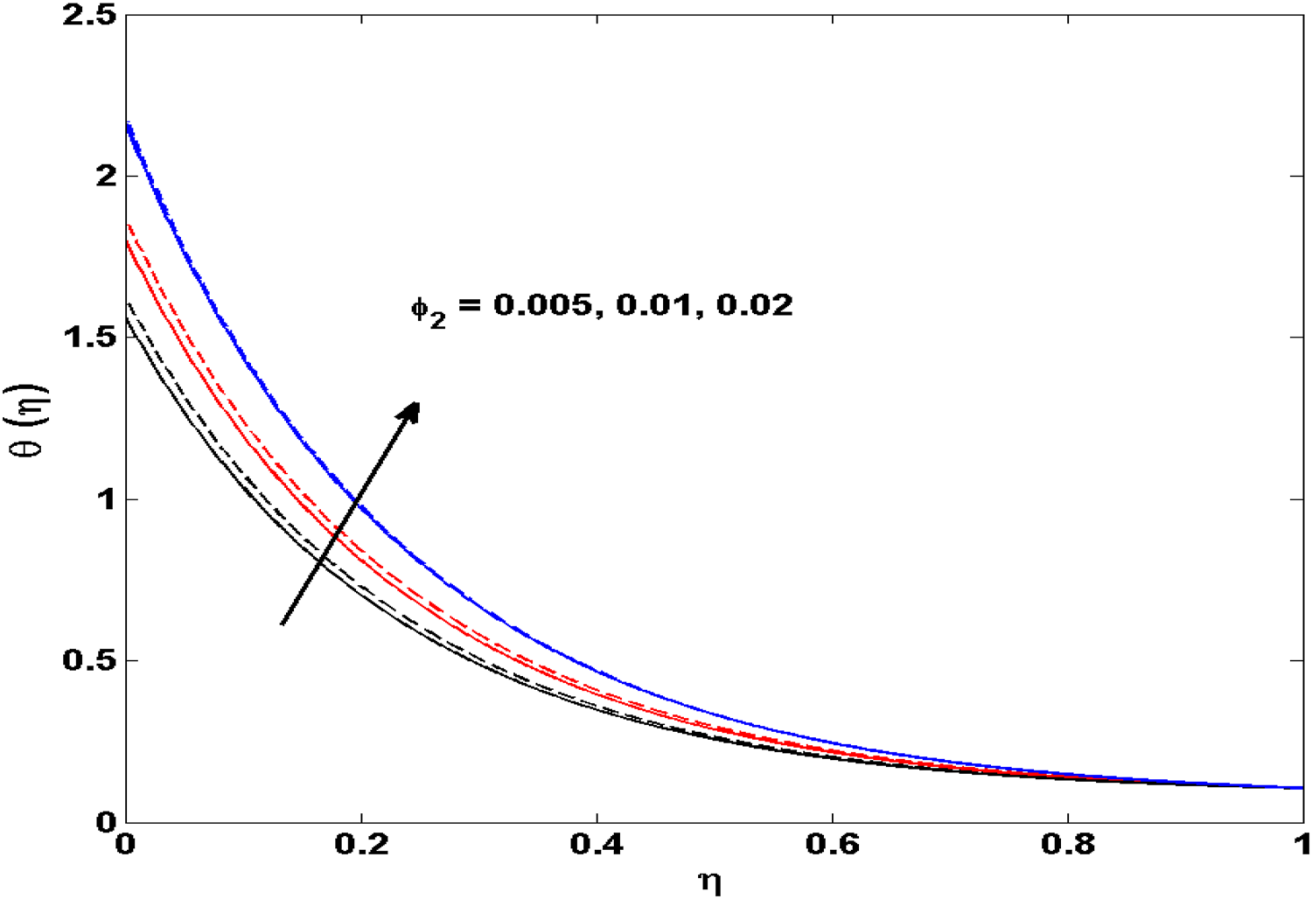
Behaviour of $\theta (\eta )$ vs $\phi_{2}$. Image generated by using MATLAB 2015a https://www.mathworks.com/help/simulink/release-notes-R2015a.html.

**Figure 6:**
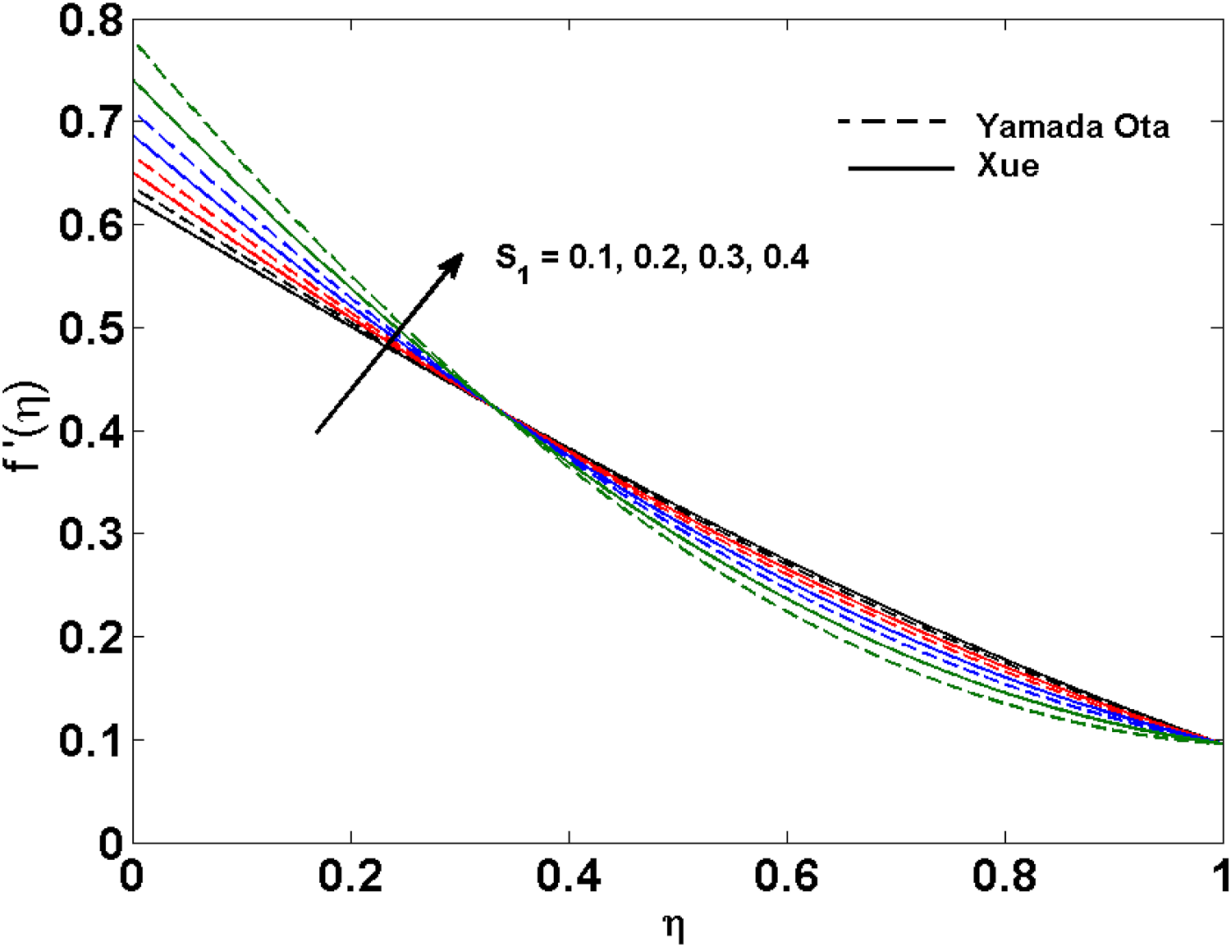
Behaviour of $f^{\prime}(\eta )$ for $S_{1}$. Image generated by using MATLAB 2015a https://www.mathworks.com/help/simulink/release-notes-R2015a.html.

**Figure 7:**
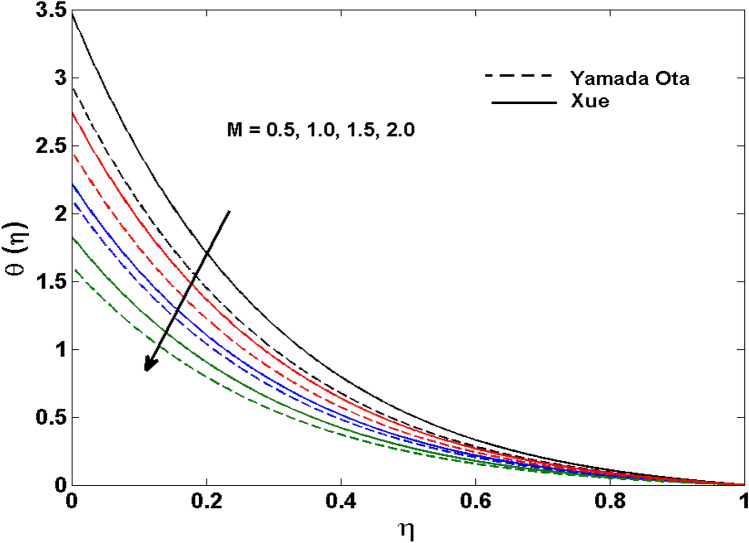
Behaviour of $\theta (\eta )$ vs $M$. Image generated by using MATLAB 2015a https://www.mathworks.com/help/simulink/release-notes-R2015a.html.

**Figure 8:**
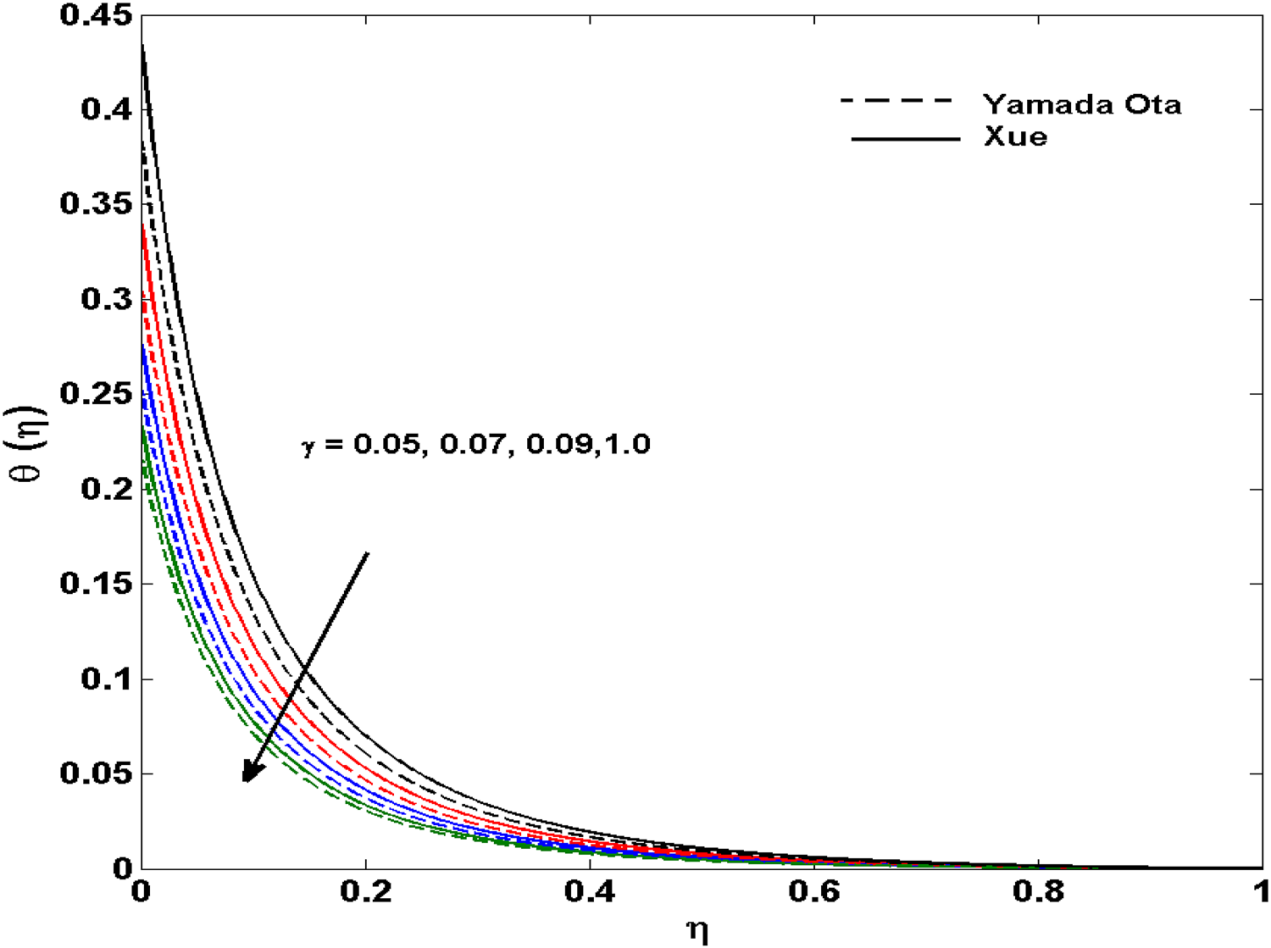
Behaviour of $\theta (\eta )$ vs $\gamma$. Image generated by using MATLAB 2015a https://www.mathworks.com/help/simulink/release-notes-R2015a.html.

**Figure 9:**
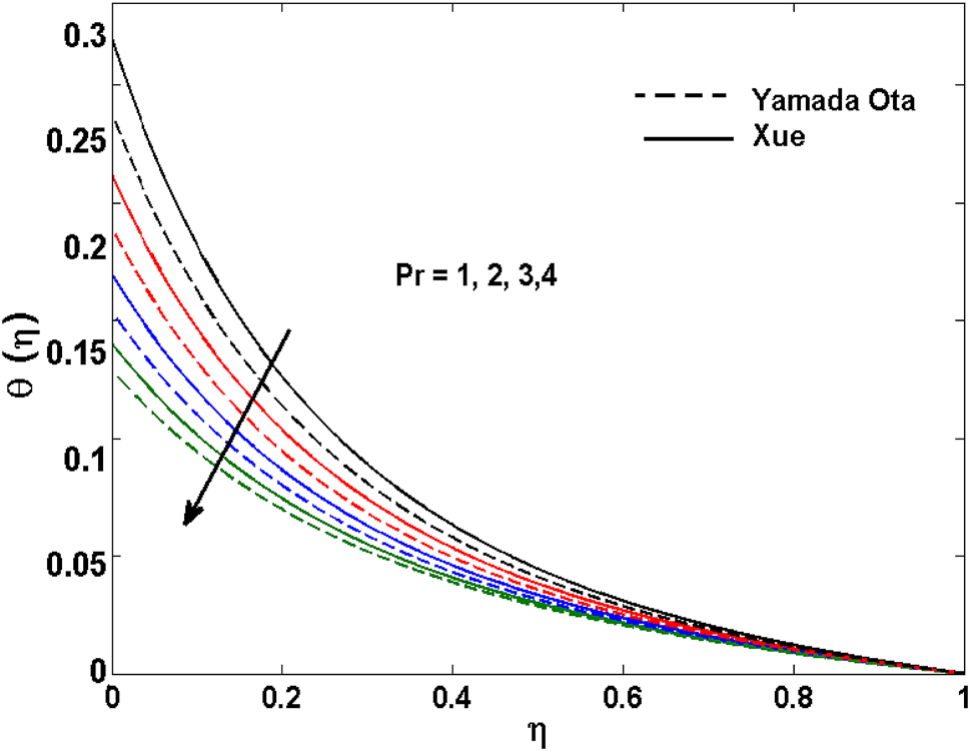
Behaviour of $\theta (\eta )$ vs $\Pr$. Image generated by using MATLAB 2015a https://www.mathworks.com/help/simulink/release-notes-R2015a.html.

**Figure 10:**
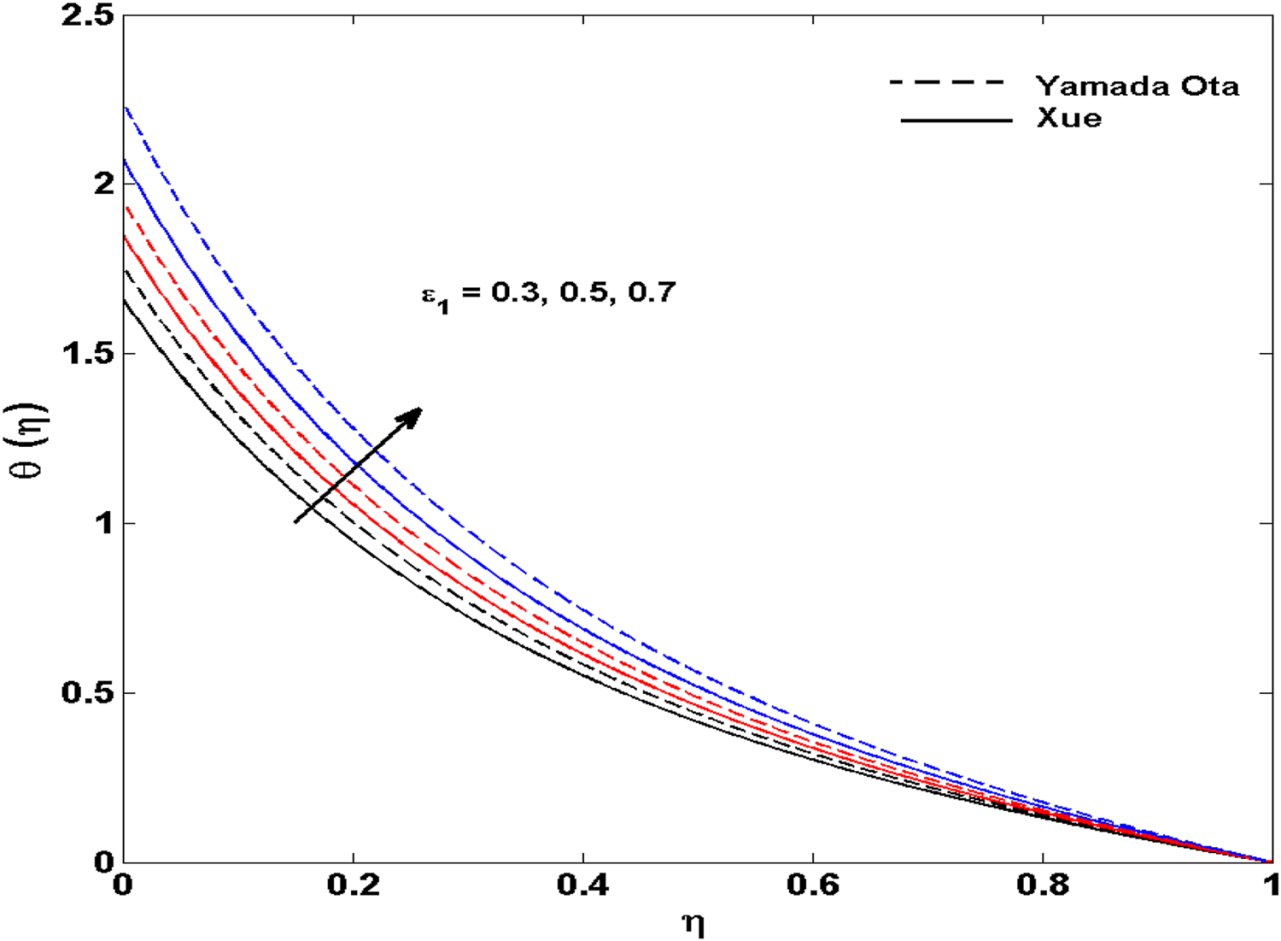
Behaviour of $\theta (\eta )$ vs $\varepsilon_{1}$. Image generated by using MATLAB 2015a https://www.mathworks.com/help/simulink/release-notes-R2015a.html.

**Figure 11:**
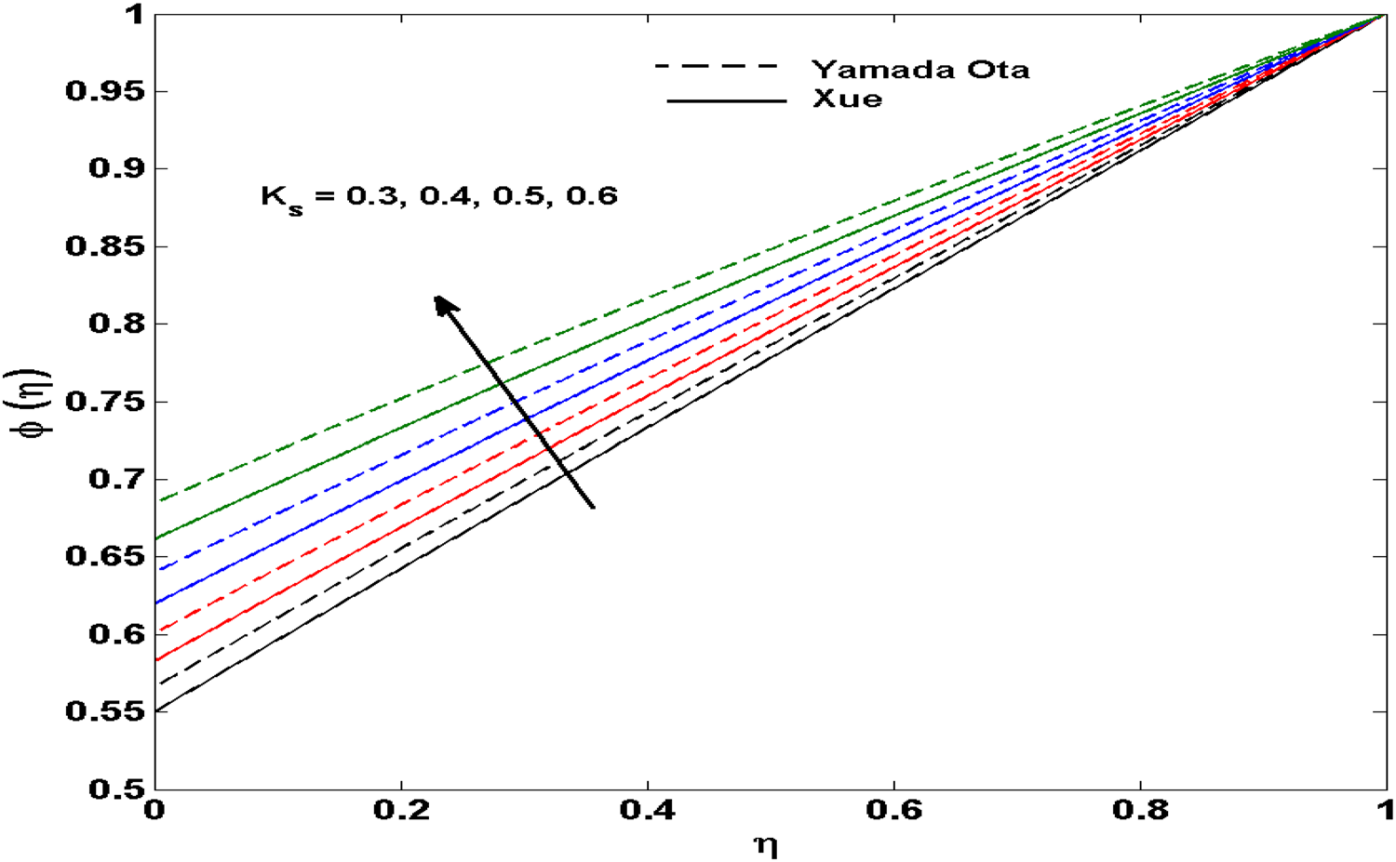
Behaviour of $\phi (\eta )$ vs $K_{s}$. Image generated by using MATLAB 2015a https://www.mathworks.com/help/simulink/release-notes-R2015a.html.

**Figure 12:**
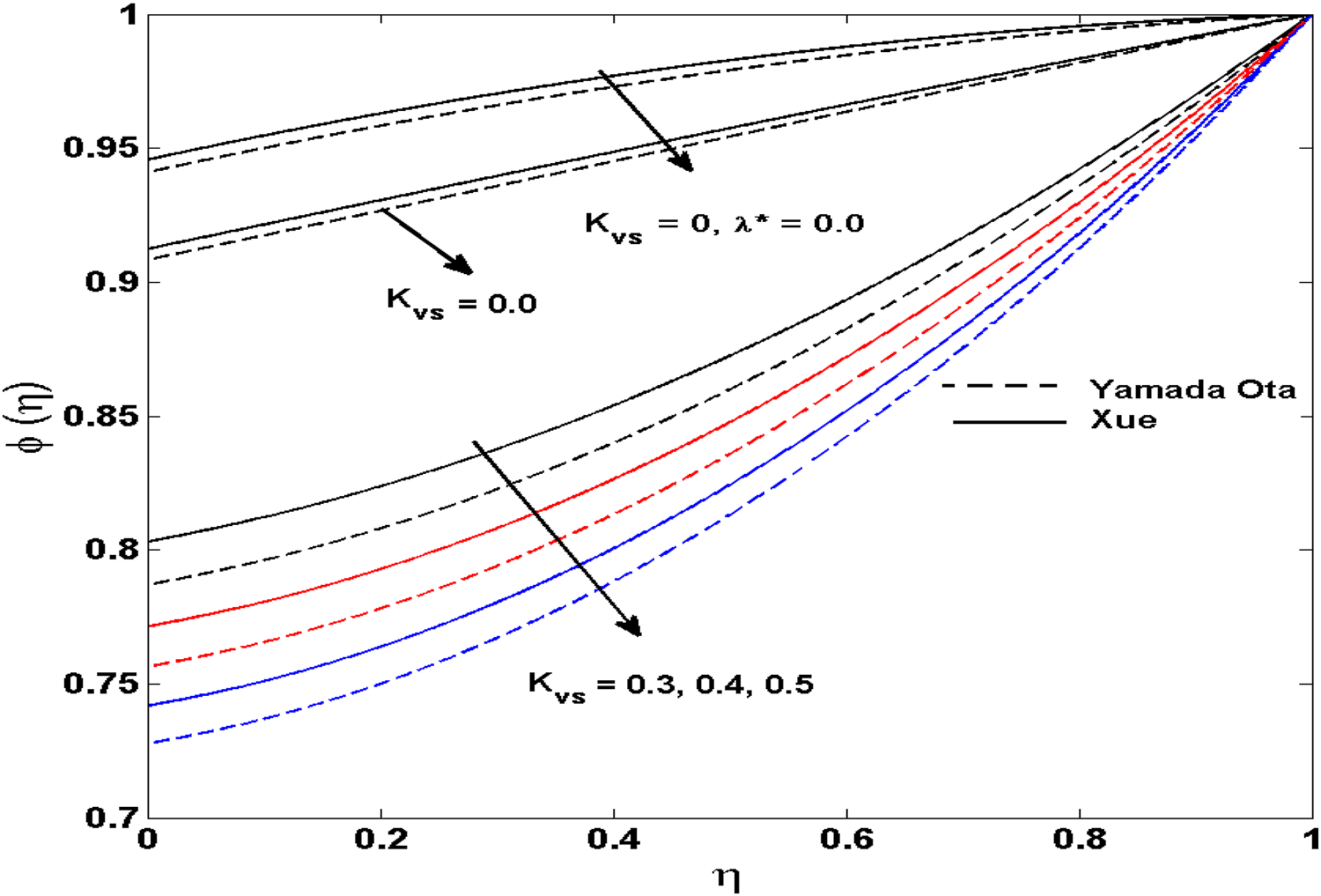
Behaviour of $\phi (\eta )$ vs $K_{vs}$. Image generated by using MATLAB 2015a https://www.mathworks.com/help/simulink/release-notes-R2015a.html.

**Figure 13:**
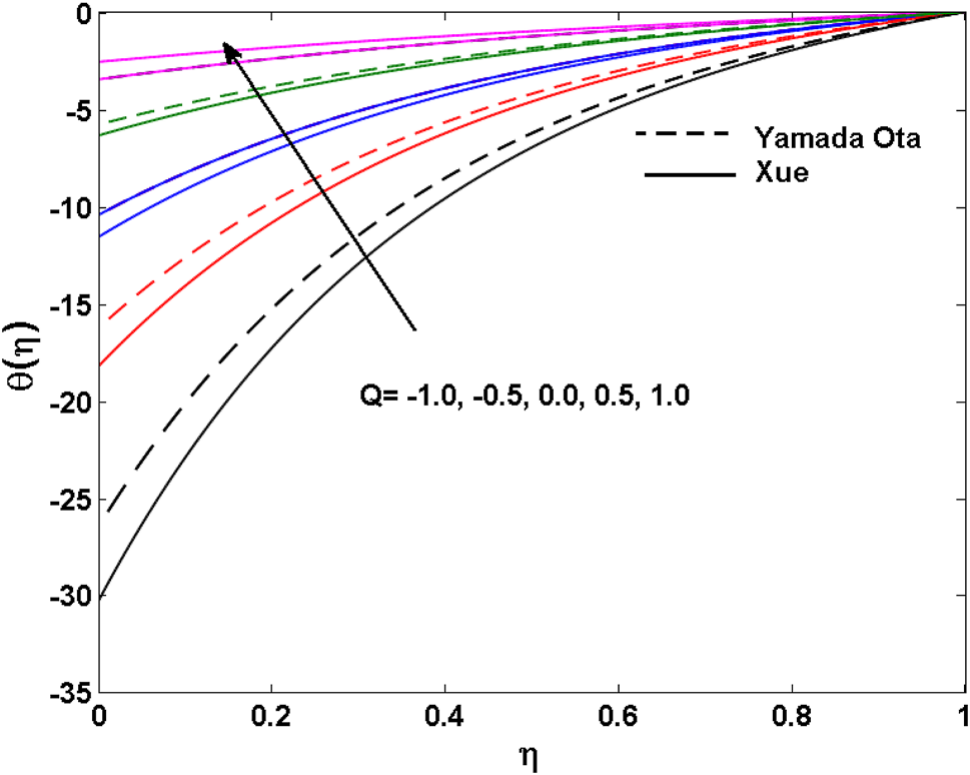
Behaviour of $\theta (\eta )$ vs $Q$. Image generated by using MATLAB 2015a https://www.mathworks.com/help/simulink/release-notes-R2015a.html.

**Figure 14:**
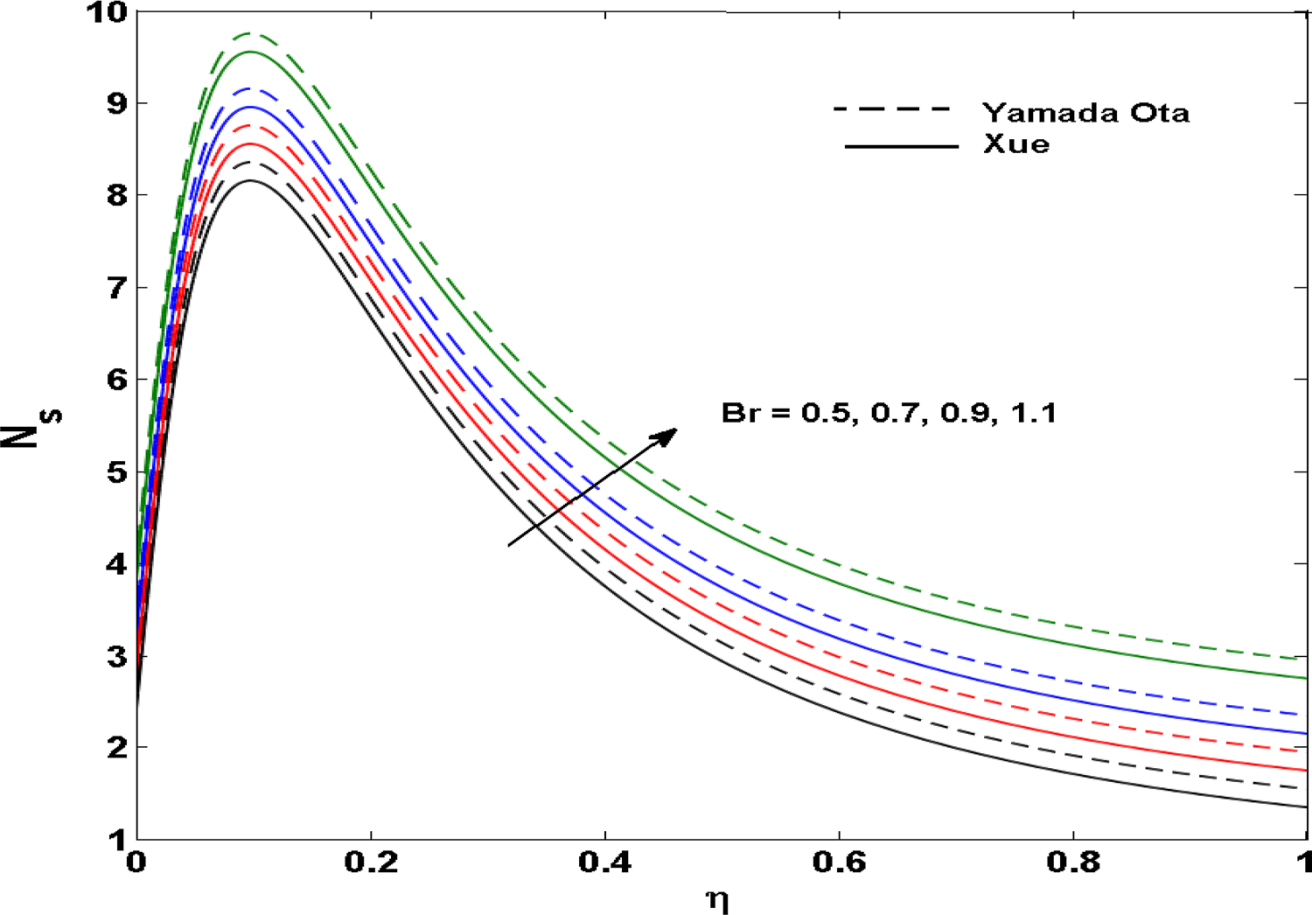
Behaviour of $Ns$ vs $Br$. Image generated by using MATLAB 2015a https://www.mathworks.com/help/simulink/release-notes-R2015a.html.

**Figure 15:**
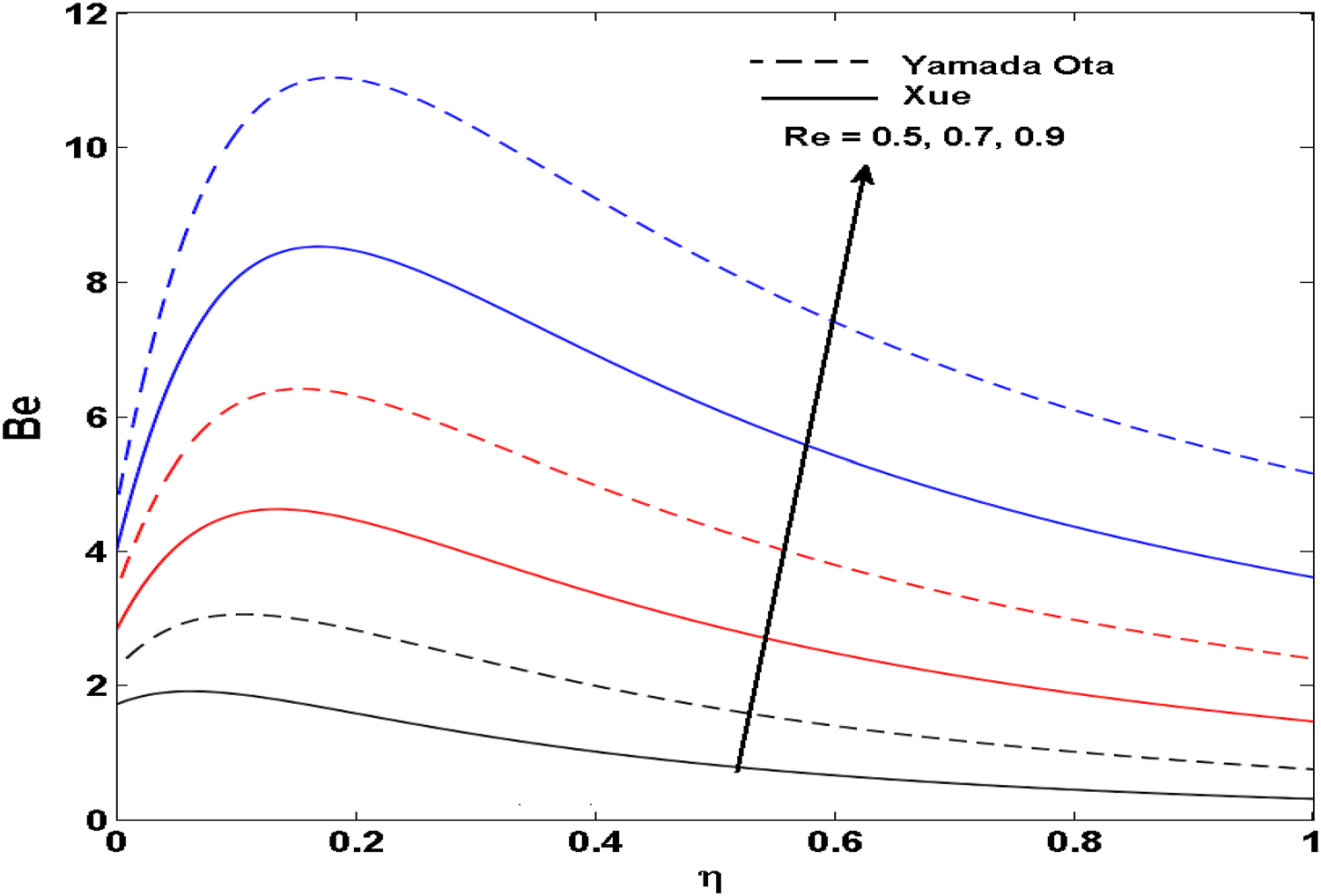
Behaviour of $Be$ vs ${\text{Re}}$. Image generated by using MATLAB 2015a https://www.mathworks.com/help/simulink/release-notes-R2015a.html.

**Figure 16:**
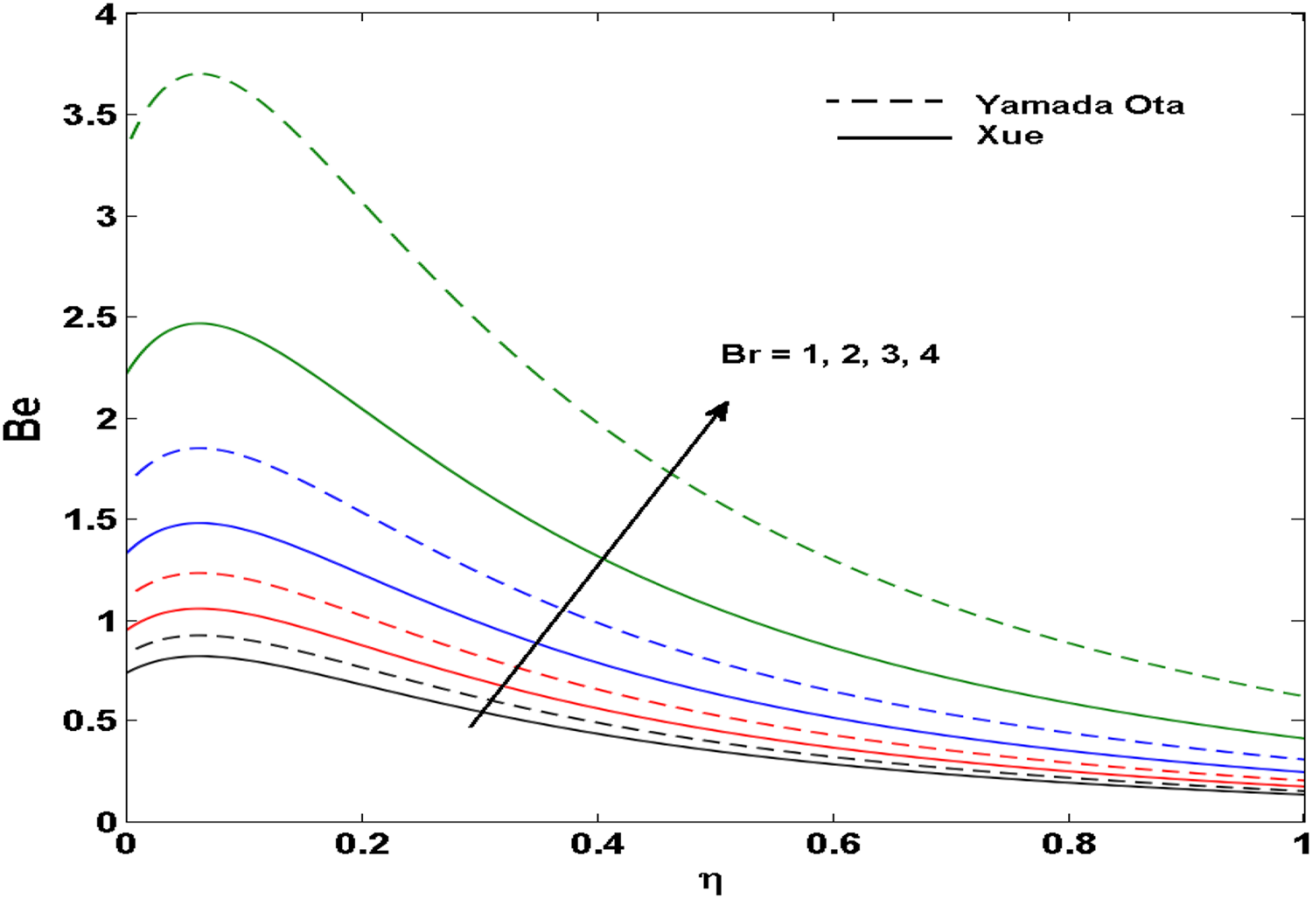
Behaviour of $Be$ vs $Br$. Image generated by using MATLAB 2015a https://www.mathworks.com/help/simulink/release-notes-R2015a.html.

**Figure 17:**
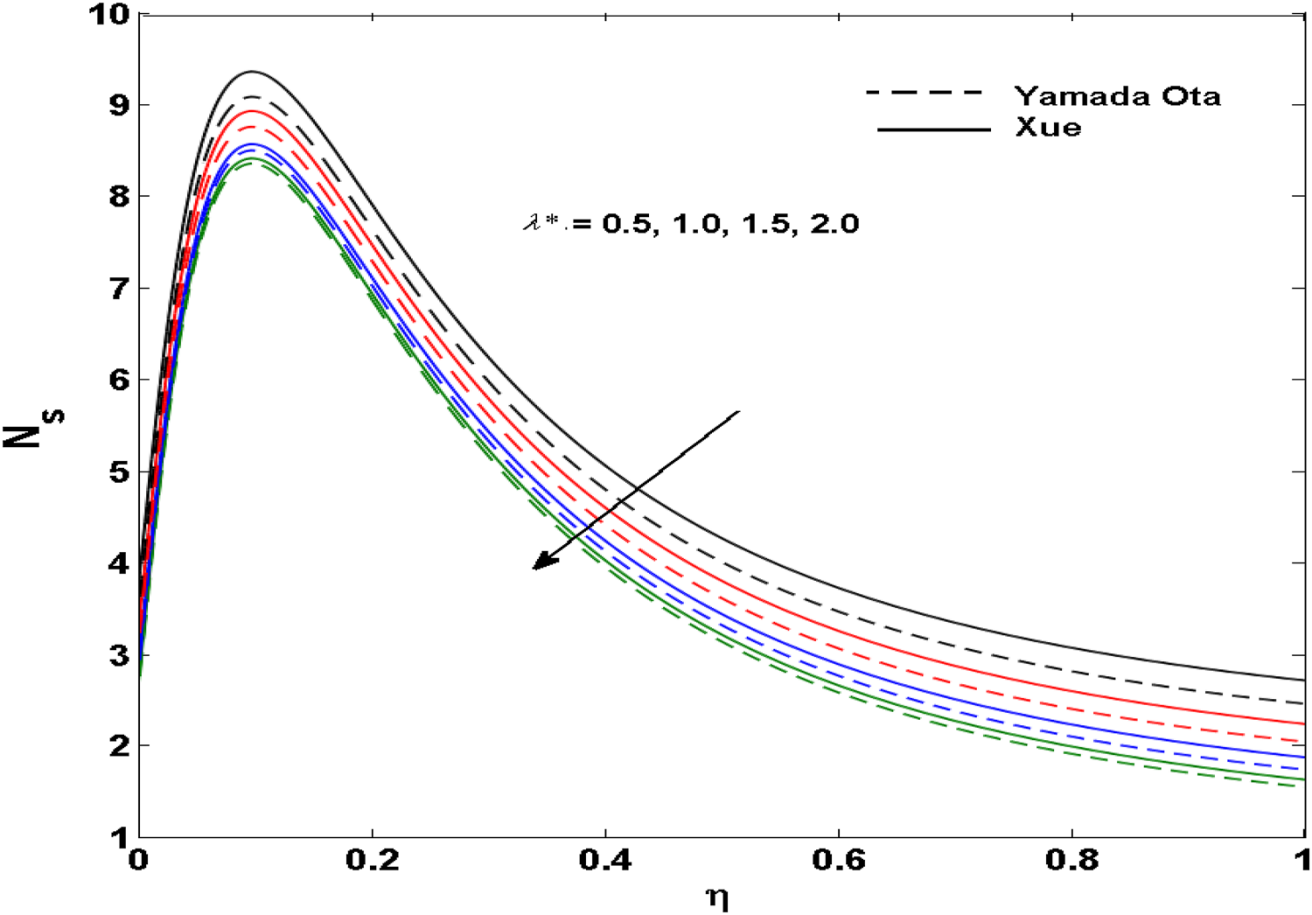
Variation of $Ns$ vs $\lambda *$. Image generated by using MATLAB 2015a https://www.mathworks.com/help/simulink/release-notes-R2015a.html.

**Figure 18:**
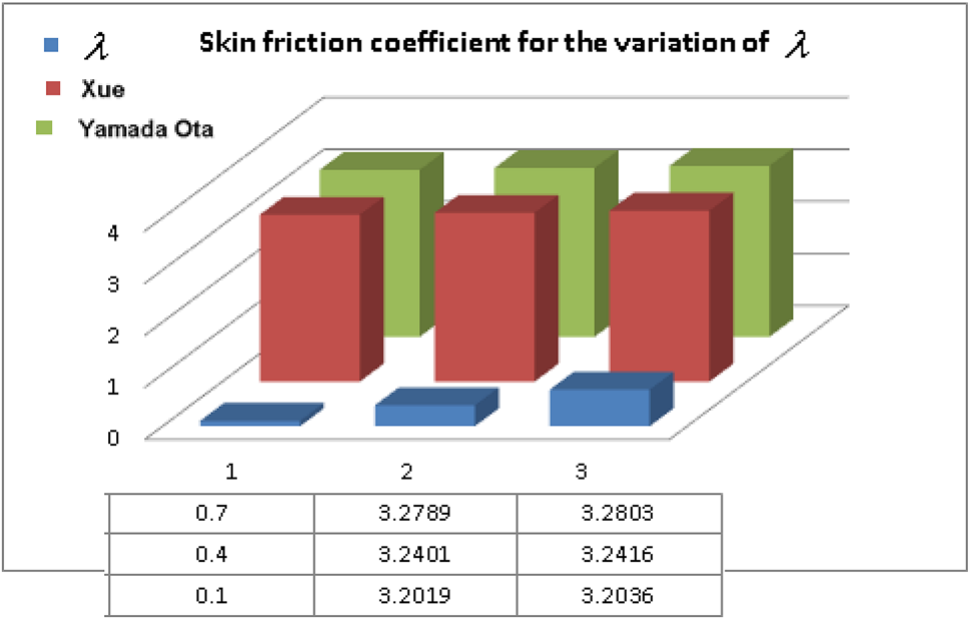
$F^{\prime\prime}(0)$ for $\lambda$_._ Image generated by using MATLAB 2015a https://www.mathworks.com/help/simulink/release-notes-R2015a.html.

**Figure 19:**
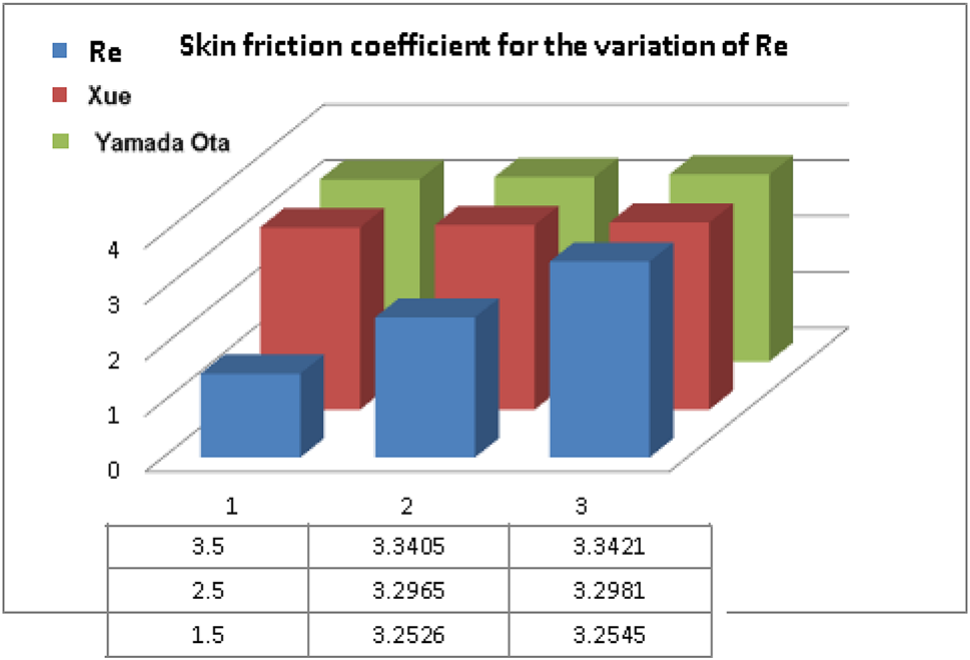
$F^{\prime\prime}(0)$ for ${\text{Re}}$_._ Image generated by using MATLAB 2015a https://www.mathworks.com/help/simulink/release-notes-R2015a.html.

**Figure 20:**
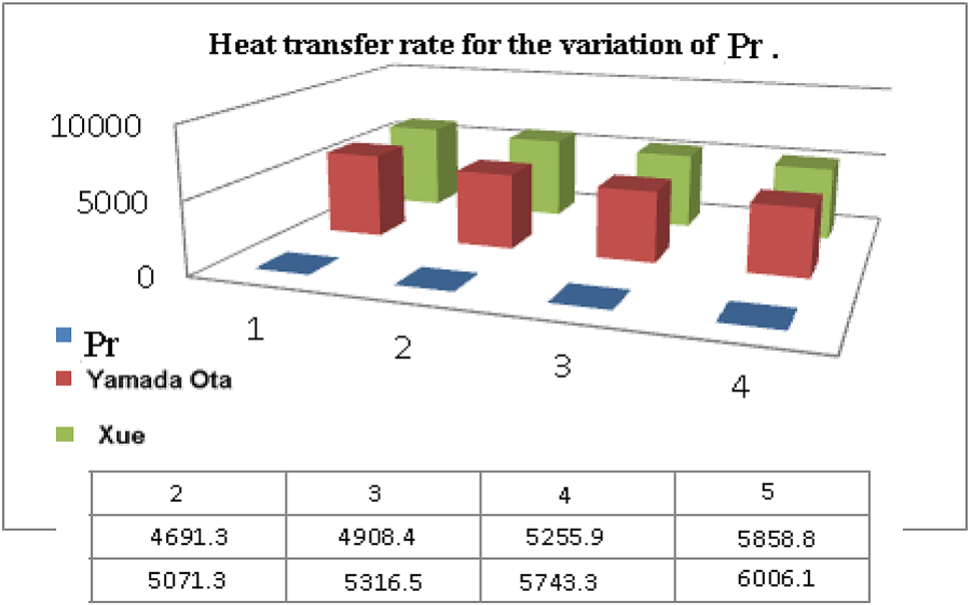
Heat transfer rate for the variation of $\Pr$_._

The Xue and Yamada–Ota are two hybrid nanoliquid models on the velocity, thermal, and concentration profiles are debated here versus the arising parameters slip parameter $\left( {0.1 \le S_{1} \le 0.4} \right),$ rotation parameter $\left( {0.5 \le \Omega \le 2.0} \right)$, Reynolds number $\left( {0.5 \le {\text{Re}} \le 3.5} \right)$, multi-wall nanoparticles volume fraction $\left( {0.005 \le \phi_{2} \le 0.02} \right),$ thermal slip parameter $\left( {0.5 \le M \le 2.0} \right)$, thermal relaxation parameter $\left( {0.5 \le M \le 2.0} \right)$, Prandtl number $\left( {1.0 \le \Pr \le 4.0} \right)$, thermal conductivity parameter $\left( {0.3 \le \varepsilon_{1} \le 0.7} \right),$ surface-catalyzed parameter $\left( {0.3 \le K_{VS} \le 0.5} \right),$ heterogeneous parameter $\left( {0.3 \le k_{s} \le 0.6} \right),$ heat generation absorption coefficient $\left( { - 1.0 \le Q \le 1.0} \right),$ porosity parameter $\left( {0.5 \le \lambda * \le 2.0} \right),$ and Brinkman number $\left( {0.5 \le Br \le 4.0} \right).$ In Fig. 2, the influence of rotation parameter ($\Omega$) on velocity profile $g(\eta )$ is presented. It is observed that for greater estimations of $\Omega$, $g(\eta )$ reduces. It is interesting to notice that the problem is reduced to 2-dimensional flow in a channel when the rotation parameter is not considered. The association of the Reynolds number (*Re*) with the velocity profiles $g(\eta )$ and $f^{\prime}(\eta )$ is portrayed in Figs. 3 and 4 respectively. A declining trend is seen for augmented Reynolds number (*Re*) estimates. Reynolds number (*Re*) is a dimensionless number that is used in various fluid flow situations to measure the flow pattern. The fluid flow appears to laminar at low *Re*, while at high *Re*, the flow of liquids tends to be turbulent. This trend of the fluid velocities versus the higher Reynolds number is according to the physics of the problem. Figure 5 is plotted for thermal profile against the growing values of nanoparticles volume fraction $\left( {\phi_{2} } \right)$ of multi-walled CNTs. It is understood that thermal profile upsurges when estimations of $\phi_{2}$ are improved. This is because of the fact that by growing the $\phi_{2} ,$ thermal conductivity upsurges and the thermal boundary layer rises. The influence of the velocity slip parameter ($S_{1}$) on the velocity field is given in Fig. 6. It is comprehended that the velocity is at its highest near the surface and it gradually diminishes as it moves away from the surface. The existence of velocity slip within the boundary layer affects the liquid velocity along the sheet to drop, as seen in this diagram. The influences of the thermal slip parameter on the thermal profile of are illustrated in Fig. 7. For higher thermal slip parameter ($M$) values, the temperature profile decays. Owing to the thermal slip increase in fluid friction with the surface is witnessed which eventually surges the fluid temperature. Furthermore, the dominance of the Yamada-Ota hybrid nanofluid model over the Xue model is obvious here. Figure 8 is shown to witness the relationship between the thermal relaxation parameter ($\gamma$) and the thermal profile. It is noticed that the thermal profile reduces for $\gamma$. In fact, the material particles take excessive time to shift the heat to the adjacent particles for large $\gamma .$ Actuality, the material possesses a non-conducting trait for large $\gamma$, thus lowering the fluid temperature. Figure 9 is sketched to see the impact of increasing Prandtl number ($\Pr$) versus thermal profile. It is envisioned that as $\Pr$ increases, thermal profile reduces. Because the thermal boundary layer thickness reduces for higher $\Pr$. Figure 10 is sketched to see the impact of increasing thermal conductivity parameter ($\varepsilon_{1}$) versus thermal profile. It is visualized that as $\varepsilon_{1}$ increases, temperature profile enhances. Physically more amount of heat is being transferred from the surface to the fluid. Consequently, the escalating trend is seen. As we move far away from the surface the thermal conductivity reduces to constant thermal conductivity and hence stops the more increase in the temperature profile. Figure 11 is sketched to address the influence of the heterogeneous parameter ($K_{s}$) on the concentration field. It is perceived that the concentration field increases with large estimates of $K_{s}$. This impression happens only in the neighborhood of the wall. Figure 12 illustrated the impact of various estimations of the surface-catalyzed parameter ($K_{vs}$) on the concentration profile. Outcomes specify that an augmentation in the $K_{vs}$ declines the concentration. The boundary layer of concentration becomes denser due to the escalation in $K_{vs}$. Actually, the rate of surface-catalyzed reaction speeds up with the increase of interface reaction on permeable media, and the rate of mass of species *A* slowly reaches the lowest at a similar position with constant $\eta$. Compared with the porous media (non-catalytic) ($\lambda * = 1,\,\,K_{vs} = 0$), the concentration of *A* decreases on the permeable media. This demonstrates the permeable media composed of the same catalyst as the sheet enormously reduces the reaction time. It is observed that when $\lambda * = 0$, physically show that permeable media is absent there, and at this stage, the concentration of species *A* is higher. It is pertinent to mention here that the influence of the Yamada-Ota hybrid nanofluid model is more dominating than the Xue model. Figure 13 is sketched to address the influence of the heat generation absorption coefficient ($Q$) on the concentration field. It is perceived that the temperature and relevant thickness have been increases with large estimates of $Q$. It is observed that when more heat is produced due to Q that is why the temperature field is enlarged. Figures 14, 15, 16 and 17 are depicted to observe the impact of evolving parameters on entropy generation *Ns* and Bejan number (*Be*) for both Yamada-Ota and Xue hybrid nanofluid models. In general, the rate of entropy generation is greater for Yamada-Ota than the Xue model. Figure 14 shows the effect of Brinkman number (*Br)* on *Ns.* It is delineated that with an escalation in *Br*, the entropy generation rate increases. Furthermore, an increase in entropy generation rate, caused by friction of fluid and by the mounting values of *Br* Joule dissipation occurs. The effect of Reynolds number (*Re)* on *Be* is expressed in Fig. 15. *Be* increases for high Reynolds number. Due to the increase in *Re* disordered motion occurs and the fluid starts moving more quickly and thus ends up contributing to heightened fluid friction and heat transfer rate affecting the entropy to escalate. Figure 16 shows the effect of *Br* on Be. For greater values of *Br,* the Bejan number reduces. For $Br = 0,$ viscous dissipation, irreversibility disappears and only heat transfer irreversibility produce. Higher estimates of porosity parameter ($\lambda *$) cause a decrease in entropy generation rate shown in Fig. 17. Tables 2, 3 and Figs. 18 and 19 display the effects of drag force coefficient for the variation of ${\text{Re}}$ and $\lambda$. It is noticed that for higher estimation of ${\text{Re}}$ and $\lambda$ drag force is rising. It is also noted that the drag force is superior for the Yamada-Ota model than for the Xue model. Here, the Yamada-Ota model attains a faster heat transfer rate than the Xue model. Table 4 and Fig. 20 display the effects of heat transfer rate for the variation of $\Pr$. It is observed that for higher estimation of $\Pr$ heat transfer rate is increases.

**Table 4:** Numerical result of $Nu_{x} \sqrt {{\text{Re}}_{x} }$ for $\Pr$.

*Pr*	$Nu_{x} \sqrt {{\text{Re}}_{x} }$	
	_Xue model_	_Yamada-Ota model_
2	4691.3	5071.3
3	4908.4	5316.5
4	5255.9	5743.3
5	5858.8	6006.1

**Figure 21:**
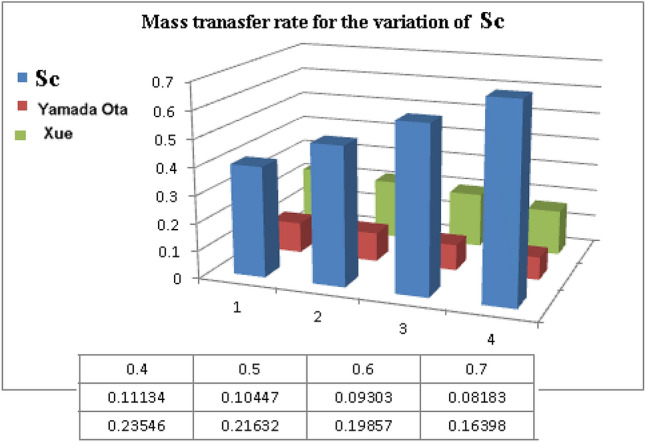
Mass transfer rate for the variation of $Sc$_._

Table 5 and Fig. 21 display the effects of mass transfer rate for the variation of Schmidt number $Sc$. It is noticed that for higher estimation of $Sc$ mass transfer rate is reduces.

**Table 5:** Numerical estimation of $Sh_{x} \sqrt {{\text{Re}}_{x} }$ for $Sc$.

$Sc$	$Sh_{x} \sqrt {{\text{Re}}_{x} }$	
	_Xue model_	_Yamada-Ota model_
0.4	0.11134	0.23546
0.5	0.10447	0.21632
0.6	0.09303	0.19857
0.7	0.08183	0.16398

## Final remarks

In this study, we have examined the hybrid nanofluid flow (SWCNTs-MWCNTs/Ethelyn glycol) with Yamada-Ota and Xue model in a rotating channel with C–C heat flux. The heat source/sink, slip effects, with the surface catalyzed reaction amalgamated with HOM-HET chemical reactions are also considered. The entropy generation analysis is also considered for the said hybrid nanofluid flow in the specific geometry. The problem is addressed numerically. The noteworthy observations of the model are appended as given below:

- The fluid velocity is diminished for the higher rotation while an opposing trend is witnessed for the strong slip.
- Higher estimations of the surface-catalyzed parameter cause reduction in the fluid concentration. However, the temperature profile is reduced for the thermal relaxation parameter.
- The dominance of the Yamada-Ota hybrid nanofluid model is obvious in comparison to the Xue model.
- Large estimates of the Reynolds number and porosity parameter affect the entropy generation rate.
- The heat and mass transfer rates increase and decrease for large estimates of the Prandtl and Schmidt numbers respectively.

## List of symbols

$$a_{0} ,e,a,b$$: Dimensional constant
$M$: Thermal slip parameter
$$s_{1}$$: Slip coefficient
$L$: Length of the nanoparticles
$k_{HNF}$: Thermal conductivity of nanofluid
*T*_*h*_: Temperature on wall
$$Br$$: Brinkman number
$S$: Velocity slip parameters
$Q$: Heat gen/abs parameter
$\mu_{HNF}$: Viscosity of hybrid nanofluid
$$T$$: Liquid temperature
*U*_*w*_: Stretching velocity
$$\phi_1, \phi_2$$: Nanoparticles volumetric
$A*$: Diffusion coefficient of chemical specie
$$Sc$$: Schmidt number
$k{}_{bF}$: Thermal conductivity (base fluid)
$$HNF$$: Hybrid nanoliquid
$\varepsilon_{2} ,\varepsilon_{1}$: Diffusion parameter and Thermal conductivity
$\gamma$: Thermal relaxation parameter
$$\lambda$$: Porosity parameter
$$\Omega$$: Angular velocity
$\Omega_{2}$: Temperature ratio parameter
$$\nu$$: Kinematic viscosity
$K_{vs}$: Surface catalyze reaction
$k$: Chemical reaction parameter
$\rho_{{s_{1} }} ,\rho_{{s_{2} }}$: Density of nanoparticles
$$\lambda$$: Permeability parameter
$Ns$: Total entropy generation
$k_{s}$: Coefficient of heterogeneous reaction
$$k*$$: Porosity coefficient
$Q_{0}$: Heat generation/absorption coefficient
*C*_*f*_: Drag force
$$A_{0}$$: Uniform suction
$R$: The radius of the nanoparticles
$(\rho c_{p} )_{HNF}$: The ratio of heat capacity
Pr: Prandtl number
$\varepsilon$: Suction/injection parameter
*Re*: Local Reynolds number
$k_{1}$: Chemical reaction parameter
$T$: Viscous stress tensor
$\rho_{HNF}$: Density of hybrid nanofluid
$${\text{Re}}$$: Reynold’s number
$$F$$: Fluid
$(u,v,w)$: Components of velocities
*T*_*0*_: Diffusive temperature
$$\nu_{f}$$: Kinematic viscosity
$$D_{A}$$*,*$$D_{B}$$: Diffusion coefficients
$\kappa_{\infty }$: Free stream thermal conductivity
$K_{s}$: Heterogeneous reaction parameter
$k_{p}$: Thermal conductivity of nanoparticles
$$L_{1} ,L_{2}$$: Diffusion parameters
$\lambda_{2}$: Thermal relaxation time
$\delta$: The ratio of the diffusion coefficient
$$c_{p}$$: Specific heat
$k_{{s_{1} }} ,k_{{s_{2} }}$: Thermal conductivity of nanoparticles
$S_{0}^{\prime \prime \prime }$: Characteristic entropy generation
$$\tau_{w}$$: Shear stress
$S_{G}^{\prime \prime \prime }$: Entropy generation rate
$$Be$$: Bejan number
